# Water-modulated conformational heterogeneity underlies multiple timescales of primary charge separation in photosystem II

**DOI:** 10.1038/s41467-026-73961-w

**Published:** 2026-06-20

**Authors:** Matteo Capone, Alfy Benny, Gianluca Dell’Orletta, Gregory D. Scholes, Dimitrios A. Pantazis, Laura Zanetti-Polzi, Isabella Daidone

**Affiliations:** 1 Institute of Nanoscience - CNR, Modena, Italy; 2 Department of Chemistry, Frick Laboratory, Princeton University, Princeton, NJ USA; 3 Department of Physical and Chemical Sciences, University of L’Aquila, L’Aquila, Italy; 4 Max-Planck-Institut für Kohlenforschung, Mülheim an der Ruhr, Germany

**Keywords:** Computational biophysics, Computational chemistry, Light harvesting

## Abstract

The kinetics of charge separation in Photosystem II, initiated within the reaction center, remain debated due to spectral congestion and overlapping timescales with energy transfer. Here, by means of atomistic molecular dynamics and quantum dynamics simulations, we present a kinetic model that attributes the observed multi-exponential behavior of the primary charge separation step to water-modulated conformational heterogeneity rather than to parallel pathways involving chemically distinct intermediate radical pairs. We propose that charge separation proceeds predominantly via the ${\,{{{\rm{Chl}}}}}_{{{{\rm{D1}}}}}^{+}{\,{{{\rm{Pheo}}}}}_{{{{\rm{D1}}}}}^{-}$ intermediate radical pair, with structural fluctuations of protein and solvent, specifically dynamic water channels near the oxygen-evolving complex, governing the multi-exponential kinetics. Analytical resolution of a kinetic scheme, which also incorporates pre-equilibration within the excited-state manifold of the reaction center, yields apparent lifetimes ( < 250 fs, 386 fs, 2.7 ps) comparable with experimental data. This model reconciles previous conflicting assignments and emphasizes the role of protein-solvent dynamics in shaping ultrafast charge separation.

## Abstract (web summary)

Photosystem II converts sunlight into chemical energy via ultrafast charge separation. Here, the authors show that water molecules in protein channels drive the multi-timescale electron transfer kinetics from multiscale quantum/classical simulations.

## Introduction

**Fig. 1:**
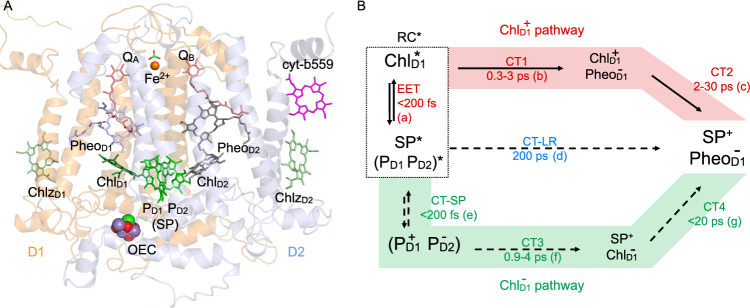
Overview of the proposed primary charge-separation pathways in the PSII reaction center. **A** Schematic structure of the PSII reaction center (RC) composed by the chain D1 (orange) and D2 (ice blue). The main cofactors of the electron transport chain are also highlighted as sticks, while the Fe^2+^ ion and the oxygen-evolving complex (OEC) cluster are shown as spheres. **B** Charge-separation scheme with all the possible pathways reported in the literature. The main proposed pathway with primary (CT1) and secondary (CT2) charge transfer after the reaction-center excitation and internal excitation energy transfer (EET) is highlighted in red as ${\,{{{\rm{Chl}}}}}_{{{{\rm{D1}}}}}^{+}$ pathway. The pathway proceeding through a ${{{{\rm{Chl}}}}}_{{{{\rm{D1}}}}}^{-}$ intermediate is highlighted in green (${\,{{{\rm{Chl}}}}}_{{{{\rm{D1}}}}}^{-}$ pathway) and comprises a CT internal to the special pair (CT-SP), a second CT forming the ${{{{\rm{SP}}}}}^{+}{{{{\rm{Chl}}}}}_{{{{\rm{D1}}}}}^{-}$ intermediate (CT3) and a secondary CT (CT4) forming the final ${{{{\rm{SP}}}}}^{+}{{{{\rm{Pheo}}}}}_{{{{\rm{D1}}}}}^{-}$ radical pair. The long-range CT (CT-LR) directly forming the ${{{{\rm{SP}}}}}^{+}{{{{\rm{Pheo}}}}}_{{{{\rm{D1}}}}}^{-}$ radical pair is reported in blue. All processes that have been proposed as minor or disfavored in some studies are shown as dashed lines. The time scales reported were proposed in (a), ^11,14,15,17,18,21,73^ (b), ^11,14,17–19,21,72,73^ (c), ^11,15,17–19,22,73^ (d), ^11,19^ (e), ^15–17^ (f), ^12,15–18^ (g)^12,15,16,72^.

Water oxidation in oxygenic photosynthesis is accomplished by Photosystem II (PSII), a large multi-subunit membrane protein complex that transduces the energy of absorbed photons into electrochemical potential^1–3^. This transduction occurs within the reaction center (RC), where excitation energy delivered by the CP43 and CP47 internal antennae and by external light-harvesting complexes is channeled into a charge-separated (CS) state; the efficiency of this process approaches unity^3–5^. Six pigments constitute the redox-active core of the RC: four chlorophyll *a* molecules, comprising the central dimer P_D1_P_D2_ (the special pair, SP) and the accessory Chl_D1_ and Chl_D2_, and two pheophytin *a* molecules, Pheo_D1_ and Pheo_D2_. These cofactors are distributed symmetrically between the D1 and D2 polypeptides (see Fig. 1A), yet electron transfer is strictly unidirectional, confined to the D1 branch^3^. The primary photochemical outcome is formation of the metastable radical pair ${{{{\rm{SP}}}}}^{+}{\,{{{\rm{Pheo}}}}}_{{{{\rm{D1}}}}}^{-}$, which marks the onset of electron transfer (ET) cascades on both sides of the RC. The oxidized SP^+^, whose reduction potential of 1.1–1.3 V vs. SHE^6^ makes it one of the strongest oxidants in biology, abstracts an electron from Tyr_Z_; the resulting Tyr_Z_ radical then drives the sequential oxidation of the Mn_4_CaO_5_ cluster, ultimately releasing molecular oxygen.

A wide variety of experimental techniques have been employed to investigate charge separation in PSII, including photon echo studies^7^, circular dichroism^8^, Stark^9^, absorption^10–18^, vibrational^18–22^, and fluorescence^23,24^ spectroscopies. Despite these extensive research efforts, the precise mechanisms remain highly debated. This is due to several factors, including: (i) the kinetic components also involving excitation energy transfer (EET) processes; (ii) spectral congestion around 680 nm, which complicates the assignment of the time-resolved spectroscopic signatures to specific chlorophylls; and (iii) charge separation potentially following multiple pathways.

A schematic of the various pathways proposed thus far, aimed at summarizing the broad and complex literature, is presented in Fig. 1B. The most debated step is associated with primary charge separation. Two main mechanisms have been proposed. Based on photon echo studies, Prokhorenko and Holzwarth^7^ were the first to suggest that the accessory chlorophyll on the D1 branch (Chl_D1_) serves as the initial electron donor. We refer to this mechanism as the ${\,{{{\rm{Chl}}}}}_{{{{\rm{D1}}}}}^{+}$ pathway, which comprises two subsequent charge transfer (CT) events, namely CT1 and CT2 (Fig. 1B):

1 $${{{{\rm{RC}}}}}^{*}\to {{{{\rm{Chl}}}}}_{{{{\rm{D1}}}}}^{+}{\,{{{\rm{Pheo}}}}}_{{{{\rm{D1}}}}}^{-}\to {{{{\rm{SP}}}}}^{+}{{{{\rm{Pheo}}}}}_{{{{\rm{D1}}}}}^{-}$$

Additional spectroscopic techniques, including transient absorption measurements with broadband visible and mid-infrared (mid-IR) probes^11,19,21^, as well as recent two-dimensional electronic (2DES) and electronic-vibrational (2DEV) spectroscopic experiments^18,22,25^, have provided support to the ${\,{{{\rm{Chl}}}}}_{{{{\rm{D1}}}}}^{+}$ pathway, specifically demonstrating that Pheo_D1_ is the primary electron acceptor. In our recent work, we also provided computational support to the ${\,{{{\rm{Chl}}}}}_{{{{\rm{D1}}}}}^{+}$ pathway and investigated the kinetics of the ${\,{{{\rm{Chl}}}}}_{{{{\rm{D1}}}}}^{+}{\,{{{\rm{Pheo}}}}}_{{{{\rm{D1}}}}}^{-}\to {{{{\rm{SP}}}}}^{+}{{{{\rm{Pheo}}}}}_{{{{\rm{D1}}}}}^{-}$ step^26^. Our results, in agreement with previous experiments^11,19^, also showed the feasibility of a direct, long-range primary CS from the excited SP to Pheo_D1_ (Charge Transfer-Long Range, CT-LR, see Fig. 1B), bypassing intermediate steps to form the metastable radical pair:

2 $${{{{\rm{RC}}}}}^{*}\to {{{{\rm{SP}}}}}^{+}{{{{\rm{Pheo}}}}}_{{{{\rm{D1}}}}}^{-}$$

Given the different time scales involved in the ${\,{{{\rm{Chl}}}}}_{{{{\rm{D1}}}}}^{+}$ pathway and CT-LR, the latter is expected to be a minor channel, the efficiency of which depends on the relative timescales of all possible competing channels, including the EET between ${\,{{{\rm{Chl}}}}}_{{{{\rm{D1}}}}}^{*}$ and SP^*^.

Through transient absorption studies, Shuvalov and colleagues ^12,16^ proposed a mechanism in analogy to the charge-separation process in purple bacterial reaction centers. In this alternative pathway, that we name ${\,{{{\rm{Chl}}}}}_{{{{\rm{D1}}}}}^{-}$ pathway, charge-separation initiates at the special pair (${\,{{{\rm{P}}}}}_{{{{\rm{D1}}}}}^{-}{{{{\rm{P}}}}}_{{{{\rm{D2}}}}}^{+}$) and proceeds with Chl_D1_ acting as the primary electron acceptor, comprising three different CT events named CT-SP, CT3 and CT4:

3 $${{{{\rm{RC}}}}}^{*}\to {{{{\rm{P}}}}}_{{{{\rm{D2}}}}}^{+}{{{{\rm{P}}}}}_{{{{\rm{D1}}}}}^{-}\to {{{{\rm{SP}}}}}^{+}{{{{\rm{Chl}}}}}_{{{{\rm{D1}}}}}^{-}\to {{{{\rm{SP}}}}}^{+}{{{{\rm{Pheo}}}}}_{{{{\rm{D1}}}}}^{-}$$

Regarding the timescales involved, charge-separation processes have been observed to comprise at least two and up to seven distinct kinetic components, ranging from sub-picoseconds to nanoseconds^7,10–20,22,27,28^. Concerning the ${\,{{{\rm{Chl}}}}}_{{{{\rm{D1}}}}}^{-}$ pathway, it has been suggested that the CS within the special pair (CT-SP, leading to ${{{{\rm{P}}}}}_{{{{\rm{D1}}}}}^{-}{{{{\rm{P}}}}}_{{{{\rm{D2}}}}}^{+}$) is rapid, occurring in less than 200 fs^12,15,17^. In contrast, other studies suggest a slow or even absent CT-SP process^22^. Consistent with these latter findings, the P_D1_^−^P_D2_^+^ charge-separated state was identified as a high-energy state in previous Quantum Mechanics/Molecular Mechanics (QM/MM) studies^29^. The subsequent formation of the ${{{{\rm{SP}}}}}^{+}{\,{{{\rm{Chl}}}}}_{{{{\rm{D1}}}}}^{-}$ radical pair has been estimated to occur on a timescale of 1–3 ps^12,14–16^. However, according to recent calculations, the CT3 step is thermodynamically unfavorable^26^.

Concerning the ${\,{{{\rm{Chl}}}}}_{{{{\rm{D1}}}}}^{+}$ pathway, the time scale involved in the formation of the primary ${\,{{{\rm{Chl}}}}}_{{{{\rm{D1}}}}}^{+}{\,{{{\rm{Pheo}}}}}_{{{{\rm{D1}}}}}^{-}$ radical pair is hotly debated. Some support the hypothesis that it forms on a sub-picosecond time scale^15,18,19,21,22^, others in a few picoseconds^11,15–17,22^. By means of transient absorption spectroscopy on the D1/D2-cytb559 complex with an extended detection window of 3 ns, van Grondelle et al. proposed a reconciling scenario according to which the two pathways (namely ${\,{{{\rm{Chl}}}}}_{{{{\rm{D1}}}}}^{+}$ and ${\,{{{\rm{Chl}}}}}_{{{{\rm{D1}}}}}^{-}$) work in parallel with time scales of approximately 500-700 fs and 2-3 ps^14^. The need of two parallel pathways to rationalize the experimental kinetic scenario was subsequently restated^15^, but the assignment of kinetic constants to specific processes proved to be non-straightforward.

Controversies also exist regarding the time scale of the secondary CS steps which, independently of the nature of the primary radical pair, lead to the formation of the ${{{{\rm{SP}}}}}^{+}{\,{{{\rm{Pheo}}}}}_{{{{\rm{D1}}}}}^{-}$ metastable radical pair^11,14,16,19,30^. Formation of this pair has been assigned timescales ranging from few ps^11,18–20^ to several tens of ps^12,14,17,31^. Additionally, late formation of the metastable radical pair, due to EET from peripheral chlorophylls^11,21,22^, has also been proposed, with reported timescales varying widely: from a few picoseconds^32^, to tens^11,14,21^, and even up to hundreds of picoseconds^11,14,19,22^. Longer time components, on the order of hundreds of picoseconds to nanoseconds, have been also associated with the relaxation of the metastable pair in isolated reaction centers^11,14,19^ or the reduction of quinone Q_A_ by ${\,{{{\rm{Pheo}}}}}_{{{{\rm{D1}}}}}^{-}$ in intact PSII^11^. In this complex scenario, our aim in this paper is to provide a comprehensive model of the primary charge separation, based on theoretical-computational methods, and to propose a possible rationalization of recent experiments. Theoretical chemistry has provided crucial insights into the interpretation of experimental data, ranging from calculations of the redox potentials^26,33^ and excited-state properties^18,22,29,34–36^ of the chromophores in the RC to the modeling of the CS dynamics^26,37,38^.

In this work, we employ data obtained by means of a computational multi-scale approach based on the Perturbed Matrix Method (PMM), applied in conjunction with large-scale classical molecular dynamics (MD) simulations and quantum-classical (QM/MM) optimizations^26,39–43^, to reconstruct the primary charge-separation mechanism. This approach has already been reliably used to reproduce and explain the underlying molecular mechanisms of many experimental observables in complex systems, including redox potentials, side-chain pK_*a*_ values, infrared and UV-Vis absorption spectra, and electron transfer rates^26,39,41,43–46^. The strength of the methodology is that it explicitly accounts for the conformational flexibility of the environment, which has a recognized role in modulating the properties of the RC^26,29,35,47–49^. The extensive sampling of the conformational space achievable with the MD-PMM approach enables the capture of the coupling between intermolecular electron transfer and protein/solvent dynamics over long timescales. However, unlike non-adiabatic mixed quantum-classical (NA-MQC) dynamics methods, MD-PMM does not explicitly propagate the electronic wavefunction, which may become critical in systems dominated by ultrafast intramolecular relaxation processes. Moreover, it does not explicitly treat electronic coherence. In the case of the ET from Chl_D1_ to Pheo_D1_ in the reaction center of PSII, however, the process is intermolecular and is expected to be strongly modulated by protein and solvent fluctuations. Under these conditions, and given the experimentally demonstrated suppression of electronic coherence at physiological temperatures^50^, the MD-PMM approach provides an appropriate and physically justified description of the charge-transfer process. Moreover, numerically exact quantum dynamics simulations with the time-dependent density matrix renormalization group (TD-DMRG) formalism^51–54^ are used to obtain estimates for the EET inside the RC. Our global kinetic model provides rates that match multiple experimentally obtained rates. Based on this model, we propose a revised interpretation of the two parallel pathways concept, showing how charge-separation kinetics are decisively shaped by conformational heterogeneity and highlighting the key role of the solvent.

## Results

### Excitation energy transfer

According to the scheme presented in Fig. 1, there are three possible primary charge-separation processes after the photoexcitation of the RC, namely CT1, CT-LR and CT-SP/CT3. Some experimental works^15,17^ suggest that the SP^*^ to ${{{{\rm{P}}}}}_{{{{\rm{D1}}}}}^{-}{{{{\rm{P}}}}}_{{{{\rm{D2}}}}}^{+}$ step (CT-SP) is fast and occurs in a few hundred fs. In contrast, recent computational studies^29,55^ assign to the ${{{{\rm{P}}}}}_{{{{\rm{D1}}}}}^{-}{{{{\rm{P}}}}}_{{{{\rm{D2}}}}}^{+}$ charge-separated state a relatively high energy. Furthermore, our previous results^26^, in agreement with other studies^22,33^, show that the ${\,{{{\rm{Chl}}}}}_{{{{\rm{D1}}}}}^{-}$ pathway is unlikely to occur at room temperature and under standard illumination conditions due to the exceptionally high reduction potential of Chl_D1_, which makes the CT3 step thermodynamically unfavorable. Additionally, very recent higher-dimensional 2DEV measurements ^22,36^ suggest that the formation of the ${{{{\rm{P}}}}}_{{{{\rm{D1}}}}}^{-}{{{{\rm{P}}}}}_{{{{\rm{D2}}}}}^{+}$ couple may occur either on a long timescale or as a minor pathway^17,36^.

These findings suggest that we can exclude the ${\,{{{\rm{Chl}}}}}_{{{{\rm{D1}}}}}^{-}$ pathway from our analysis in the attempt to rationalize the experimentally observed rates (see Fig. 1). The relative contribution of the two remaining pathways (i.e., CT1/CT2 and CT-LR) to the overall kinetics depends not only on their respective rates but also on the timescale of EET between ${\,{{{\rm{Chl}}}}}_{{{{\rm{D1}}}}}^{*}$ and SP^*^.

**Fig. 2:**
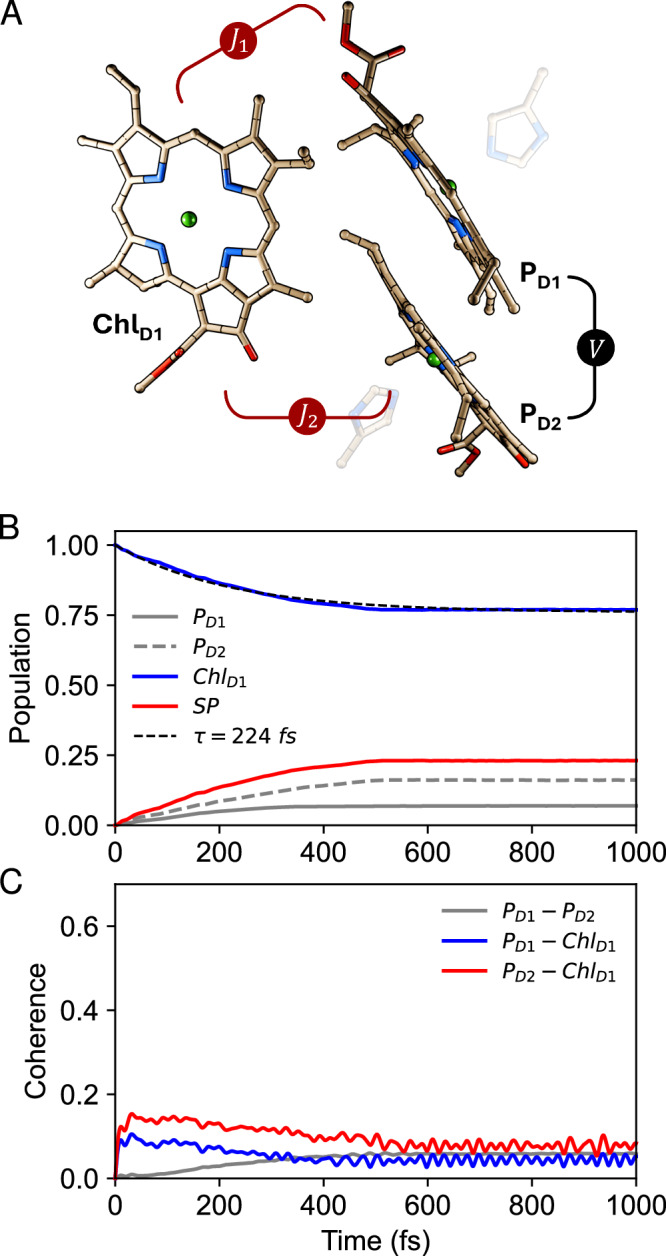
Excitation energy-transfer dynamics in the Chl_D1_-P_D1_-P_D2_ trimer from TD-DMRG quantum dynamics simulation. **A** Chl_D1_, P_D1_ and P_D2_ trimer considered for the excitation energy-transfer TD-DMRG quantum dynamics simulations using the following couplings: *V* = 150 cm^−1^, *J*_1_ = −42 cm^−1^ and *J*_2_ = −56 cm^−1^. *V*, *J*_1_ and *J*_2_ indicate P_D1_-P_D2_, P_D1_-Chl_D1_ and P_D2_-Chl_D1_ electronic couplings, respectively. **B** Excited-state populations for Chl_D1 _(blue), P_D1_ (grey solid), P_D2_ (grey dashed). Total population of P_D1_+P_D2_ is reported in red and labelled as SP. The black dashed line is the exponential fit providing the phenomenological time constant τ = 224 fs. **C** Excitonic coherence between P_D1_-P_D2_ (grey), P_D1_-Chl_D1_ (blue) and P_D2_-Chl_D1_ (red).

Energy-transfer dynamics in the P_D1_-P_D2_-Chl_D1_ trimer are governed by the characteristic excitonic couplings between the chromophores, which are tuned by intermolecular distances and relative orientations, and the coupling to the vibrational degrees of freedom. The trimer system is oriented such that the P_D1_ and P_D2_ are strongly coupled, due to the spatial proximity resulting in a combination of long-range coulombic coupling and additional short-range effects^56^. On the other hand, the larger separation along with the edge-to-face orientation between SP subunits and Chl_D1_ results in a weaker coupling between them (see Fig. 2A). To accurately include the intricate details of electron-vibrational couplings, a fully quantum-mechanical treatment of the dynamics is required. To achieve this, we carried out numerically exact quantum dynamics simulations using the time-dependent density matrix renormalization group (TD-DMRG) formalism^51–54^, with coupling between electronic and vibrational degrees of freedom incorporated via experimentally derived spectral densities, thereby ensuring a realistic description of system-bath interactions (see Suppl. Note 1)^10,57,58^. The physiological temperature of 300 K was achieved with thermo-field dynamics (TFD) by introducing auxiliary vibrational degrees of freedom^59^. In the quantum dynamics simulation we employ a set of electronic coupling parameters among the three chromophores widely used in previous studies, calculated by Renger and coworkers^21,60^:* V* = 150 cm^−1^, *J*_1_ = −42 cm^−1^ and *J*_2_ = −56 cm^−1^ for the P_D1_-P_D2_, P_D1_-Chl_D1_ and P_D2_-Chl_D1_ pairs, respectively (more data on the electronic coupling parameters are provided in Suppl. Note 1.1).

Results of the TD-DMRG simulation are shown in Fig. 2B, which reports the time-dependent populations of P_D1_, P_D2_, and Chl_D1_, with the excitation initially localized on ${\,{{{\rm{Chl}}}}}_{{{{\rm{D1}}}}}^{*}$. The results show that excitonic energy transfer between Chl_D1_ and the SP subunits occurs with a phenomenological time constant of 224 fs, without observable oscillatory components in the population dynamics. The electronic couplings from Chl_D1_ to the SP subunits (*J*_1_ and *J*_2_) are of intermediate strength, placing the transfer in a moderately coherent energy-transfer regime. Additionally, at 300 K, the effective electron-vibrational coupling, particularly in the low-frequency region, increases within the TFD formalism^59^. As a result, the coherent character of the population transfer is reduced, although not completely suppressed. The coherence measure from the numerically exact simulations reveals a gradual decay of coherence between Chl_D1_ and the SP subunits. Among these, the Chl_D1_-P_D2_ coherence is higher, owing to the slightly larger coupling. Furthermore, the strong excitonic coupling *V* between P_D1_ and P_D2_ leads to the gradual buildup of a modest but persistent coherence between them.

The population decay of ${\,{{{\rm{Chl}}}}}_{{{{\rm{D1}}}}}^{*}$ obtained from the quantum dynamics simulations was fitted using a reversible two-state kinetic model, enabling extraction of the ${k}_{{{{{\rm{Chl}}}}}_{{{{\rm{D}}}}1}\to {{{\rm{SP}}}}}$ and ${k}_{{{{\rm{SP}}}}\to {{{{\rm{Chl}}}}}_{{{{\rm{D}}}}1}}$ time constants. The extracted rates were ${k}_{{{{{\rm{Chl}}}}}_{{{{\rm{D}}}}1}\to {{{\rm{SP}}}}}={(928\,{{{\rm{fs}}}})}^{-1}$ and ${k}_{{{{\rm{SP}}}}\to {{{{\rm{Chl}}}}}_{{{{\rm{D}}}}1}}={(296\,{{{\rm{fs}}}})}^{-1}$, corresponding to the observed relaxation time of 224 fs. These results indicate efficient EET within the Chl_D1_-SP trimer.

The fast ${\,{{{\rm{Chl}}}}}_{{{{\rm{D1}}}}}^{*}/{{{{\rm{SP}}}}}^{*}$ EET kinetics, coupled to the fast CT1 kinetics (see below), also imply that the direct charge separation (CT-LR) is not a relevant channel at room temperature and standard illumination conditions. However, as previously suggested^26^, the slow CS pathway might play additional roles, either productive or protective, linked to the generation of highly oxidative species involved in water oxidation in PSII. Since the above results suggest that neither the ${\,{{{\rm{Chl}}}}}_{{{{\rm{D1}}}}}^{-}$ pathway nor the CT-LR one is the preferred pathway after RC photoexcitation, we focus in what follows on the ${\,{{{\rm{Chl}}}}}_{{{{\rm{D1}}}}}^{+}$ pathway.

### Bi-exponential behavior of the primary charge separation

**Fig. 3:**
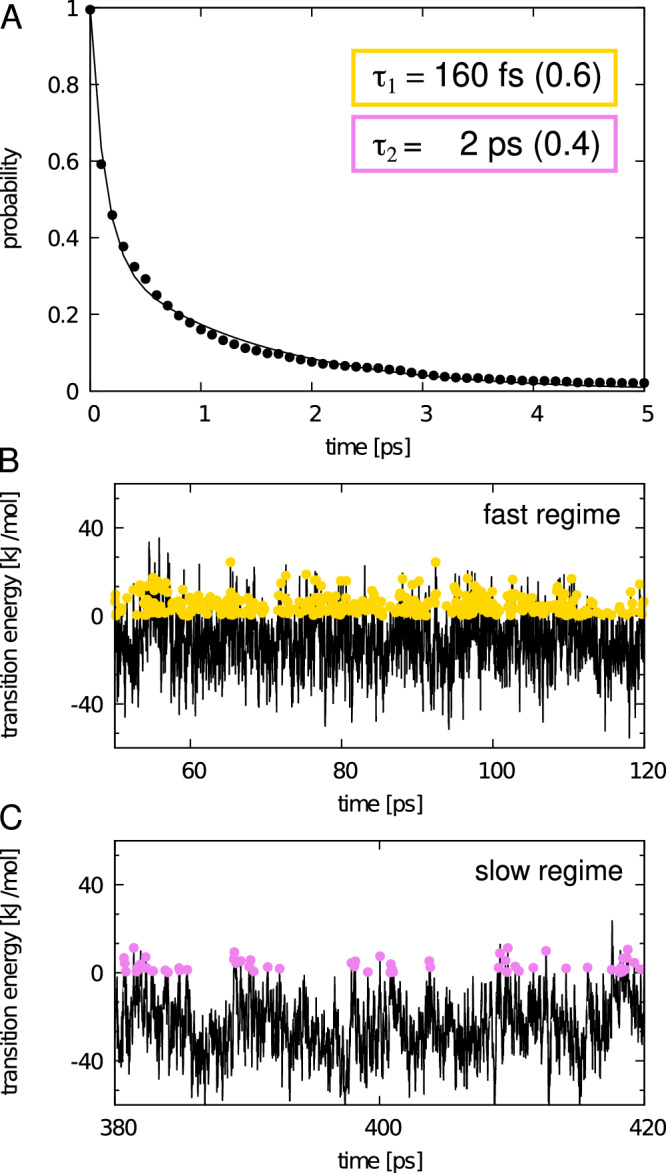
Conformational heterogeneity underlying the bi-exponential kinetics of the primary charge-separation step CT1. **A** MD-PMM kinetic profile for CT1 calculated in our previous work^26^. Note that the crossing times were scaled by the adiabatic fraction, *χ* = 0.2. The solid line is the bi-exponential fit providing for the electron transfer (ET) step *τ*_1_ = 163 fs (population of 60%) and *τ*_2_ = 2 ps (population of 40%). Portions of the transition energy TE reported in Fig. 4 exemplifying the fast (**B**) and slow (**C**) ET kinetic regimes. In the fast-ET (**B**), the instantaneous fluctuations of the TE provide a large number of points (crossing points, marked in yellow) in which the inversion of the relative energy between reactants and products occurs, leading, in the adiabatic approximation, to a CT event. In the slow regime (**C**) the crossing points (marked in purple) are considerably reduced, leading to slower kinetics.

A previous application of the MD-PMM approach to the investigation of the primary charge-separation mechanism^26^ showed a bi-exponential kinetic behavior for the primary ${{{{\rm{RC}}}}}^{*}\to {\,{{{\rm{Chl}}}}}_{{{{\rm{D1}}}}}^{+}{\,{{{\rm{Pheo}}}}}_{{{{\rm{D1}}}}}^{-}$ (CT1) step, with a fast time constant of approximately 200 fs and a slower one of 2 ps (Fig. 3). To investigate the origin of the bi-exponential kinetics for CT1, here we extend our MD simulations in the excited ${{{{\rm{Chl}}}}}_{{{{\rm{D1}}}}}^{*}$/SP state and perform the corresponding PMM calculations. This allows us to obtain a 500 ps-long trend of the transition energy (TE) associated with the CT1 reaction, that is the energy difference between products and reactants as determined from the MD-PMM calculations (see Methods section and Supplementary Note 2), given, for each *k*^*th*^ frame, by:

4 $${{{{\rm{TE}}}}}_{k}={\left[{\epsilon }_{{{{{\rm{Pheo}}}}}_{{{{\rm{D}}}}1}^{-}}+{\epsilon }_{{{{{\rm{Chl}}}}}_{{{{\rm{D}}}}1}^{+}}\right]}_{k}-{\left[{\epsilon }_{{{{{\rm{Pheo}}}}}_{{{{\rm{D1}}}}}}+{\epsilon }_{{{{{\rm{Chl}}}}}_{{{{\rm{D}}}}1}^{*}}\right]}_{k}$$

where ${\epsilon }_{{{{{\rm{Chl}}}}}_{{{{\rm{D}}}}1}^{*}}$ and ${\epsilon }_{{{{{\rm{Chl}}}}}_{{{{\rm{D}}}}1}^{+}}$ are the perturbed energies of the donor in its excited and oxidized states, respectively, and ${\epsilon }_{{{{{\rm{Pheo}}}}}_{{{{\rm{D1}}}}}}$ and ${\epsilon }_{{{{{\rm{Pheo}}}}}_{{{{\rm{D}}}}1}^{-}}$ the energies of the acceptor in its neutral and reduced states, respectively.

**Fig. 4:**
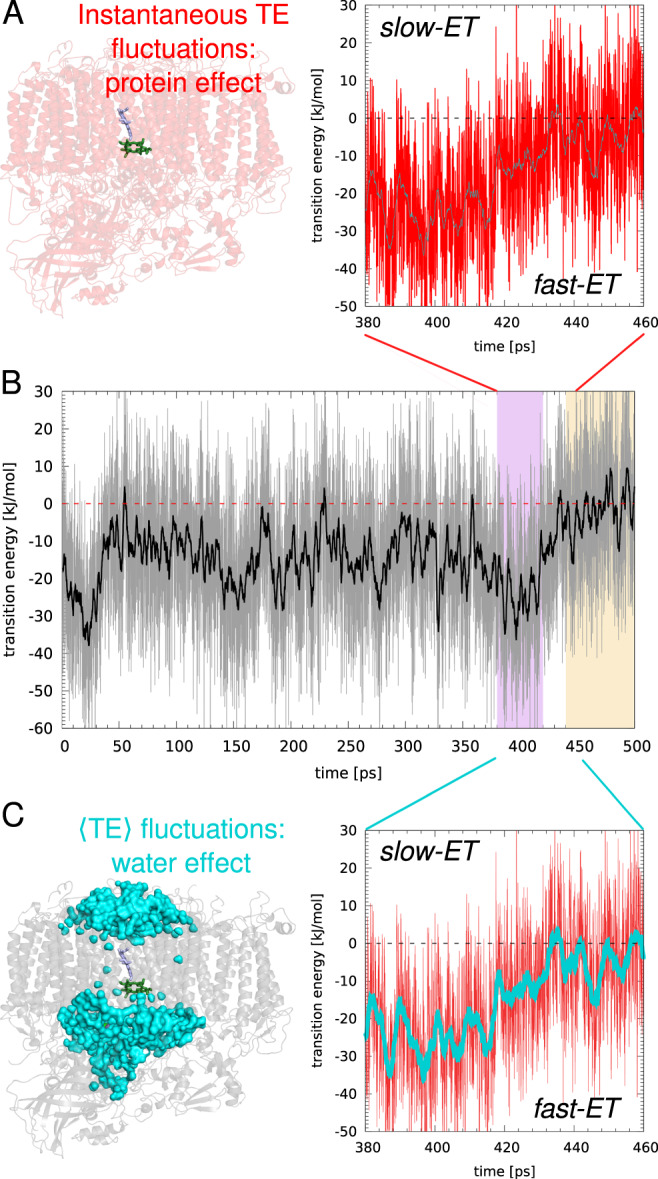
Conformational modulation of the CT1 transition energy along the non-equilibrium classical MD trajectory. The perturbed transition energy (TE) associated with CT1, calculated along the 500 ps-long classical MD simulation in the excited ${{{{\rm{Chl}}}}}_{{{{\rm{D1}}}}}^{*}$/SP state, is reported in panel **B**. The gray line reports the frame-by-frame TE, while the thick black line is the running average over 100 frames of the TE to highlight the 〈TE〉 variation. The yellow and purple areas in the graph highlight examples of the fast and slow electron transfer (ET) regimes, respectively, discussed also in Fig. 3. Panels **A** and **C** report a zoom-in of the 380–460 ps time interval, that include both fast and slow-ET regimes. In panel **A** the instantaneous fluctuations of the TE induced by the protein are highlighted as a red line. In panel **C** the 〈TE〉 is highlighted as thick cyan line, showing the transition from a slow-ET regime to a fast-ET one induced by the solvent.

The time evolution of the calculated TE, in terms of the time scale of its fluctuations, is consistent with the previously obtained bi-exponential kinetics (Fig. 4). Specifically, it can be observed that there are two distinct fluctuation regimes in the TE: instantaneous fast fluctuations around a given average TE value and slower fluctuations (on the order of tens of ps) of the average TE value (〈TE〉, highlighted as a thick black line in Fig. 4). As shown in Fig. 3B and C, the instantaneous fast fluctuations produce the inversion of the relative energy between the reactant and product states and therefore, in the adiabatic approximation, the CT event. The slower fluctuations of 〈TE〉 determine the interconversion between a fast-ET kinetic regime and a slow one, which together provide the bi-exponential kinetics.

To investigate the origin of both the instantaneous and the 〈TE〉 fluctuations, we focus on the electrostatic potential (${{{\mathcal{V}}}}$) exerted on the exchanging pigments by the environment (i.e., the protein matrix, the solvent and the counterions). As a matter of fact, ${{{\mathcal{V}}}}$ makes a major contribution to the MD-PMM perturbation operator (see Eq. (6) in Methods section and Suppl. Table 3, where the average contributions to the TE from the total electrostatic potential and from that exerted by each portion of the system are reported). In the calculation of ${{{\mathcal{V}}}}$ we neglect in what follows the contribution of the counterions. Their contribution is expected to be an artifact arising from the limited spatial and temporal sampling of the MD and, as shown in Suppl. Figure 1, the fluctuations of the electrostatic potential ${{{\mathcal{V}}}}$ calculated without the inclusion of counterions, correlate well with the TE fluctuations. Interestingly, by resolving ${{{\mathcal{V}}}}$ into contributions from the system’s main components (i.e., protein matrix, ${{{{\mathcal{V}}}}}_{p}$, and solvent, ${{{{\mathcal{V}}}}}_{s}$), we observe that the instantaneous fluctuations can be ascribed to the protein, whereas the 〈*TE*〉 fluctuations depend on the solvent. This can be appreciated in Suppl. Figure 2, where the correlation between the instantaneous and $\langle {{{\mathcal{V}}}}\rangle$ fluctuations with the fluctuations of ${{{{\mathcal{V}}}}}_{p}$ and ${{{{\mathcal{V}}}}}_{s}$, respectively, is shown.

#### Water effect

**Fig. 5:**
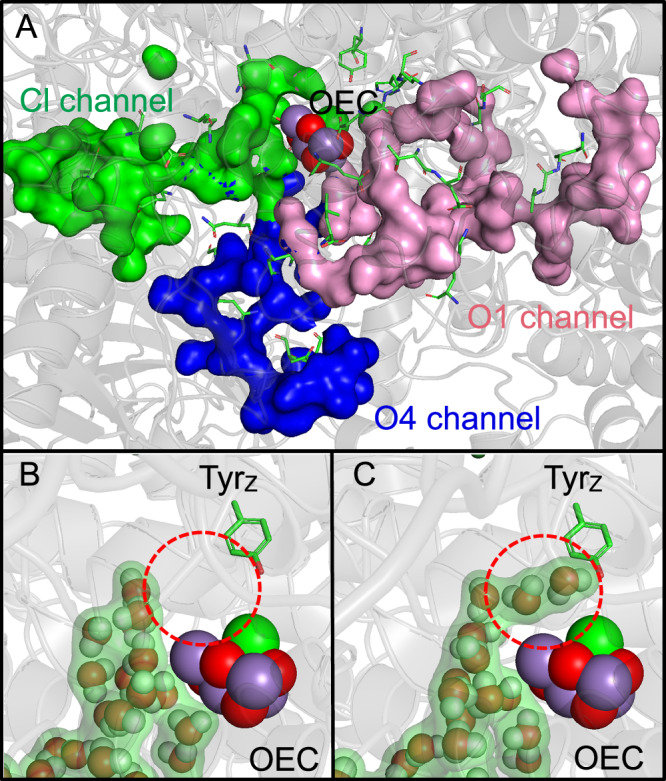
Water channels connecting bulk water to the OEC and their role in modulating the CT1 transition energy. **A** The three channels linking the external side of the PSII to the oxygen-evolving complex (OEC) are shown. The green, pink and blue surface represent the Cl, O1 and O4 channels, respectively. The OEC is depicted as Van der Waals spheres, and the residues surrounding the three channels are reported as sticks. **B**, **C** Show the extension of the Cl1 channel towards Tyr_Z_, corresponding to an increase in 〈TE〉.

Since the solvent is responsible for the exchange between the slow-ET and fast-ET regimes, the bi-exponential kinetics of CT1 are essentially due to changes in solvation. This finding is similar to previous experimental works in flavodoxins, which showed a dynamical coupling between CT kinetics and solvent relaxation^61–63^. Based on electrostatic considerations, it can be anticipated that the solvent molecules that most contribute to the 〈TE〉 fluctuations are those surrounding the exchanging pigments. Due to the position of Chl_D1_ and Pheo_D1_ within the protein matrix, the water molecules closest to the exchanging partners are internal water molecules. The presence of water channels that control the access of water to the oxygen-evolving complex (OEC; Mn_4_CaO_5_) is well known^64,65^. Three main water channels starting at the OEC and ending at bulk water have been identified: the O1 channel, the O4 channel, and the Cl1 channel (see Fig. 5A). All these channels are proposed to be important for water and proton transport.

Our data show that the electrostatic potential exerted by the internal water molecules found along the trajectory within 25 Å of Chl_D1_ and Pheo_D1_, as well as by those belonging to the OEC channels (Fig. 4C), has a correlation coefficient of 0.66 with the electrostatic potential exerted by all water molecules (see Suppl. Figure 3). These results point to a relevant role of the internal water molecules in determining the 〈TE〉 fluctuations and, consequently, the bi-exponential kinetics of CT1. Further investigation of this subset of solvent molecules shows that their effect does not depend on a change in their number (due to e.g. a change in the OEC accessibility from the bulk) but rather on their spatial organization inside the channels. This effect can be exemplified by focusing on the Cl1 channel. As shown in Suppl. Figure 4, the largest fluctuation of the TE (i.e., from ≈400 to ≈500 ps) correlates well with the time evolution of the electrostatic potential of the water molecules belonging to the Cl1 channel. Inspection of the trajectory shows that this fluctuation can be attributed to the arrangement of the molecules inside the channel (Fig. 5B). Specifically, an increase in the electrostatic potential is observed when the channel extends towards Tyr_Z_. Interestingly, Tyr_Z_ plays a crucial role along the reaction pathway, as it oxidizes the OEC cluster to complete water oxidation. Several experimental and computational studies investigated the functional role of water molecules in PSII, in particular focusing on the role of water channels starting at the OEC^64–66^, as well as on structural water molecules close to Chl_D1_^67^. In this context, our results suggest a fundamental role of solvation in two seemingly disparate functions of PSII function, namely the charge separation in the RC chromophores and the catalytic water oxidation at the OEC cluster.

#### Protein effect

To analyze the role of the electrostatic potential exerted by the protein and how it tunes the instantaneous fluctuations of the TE, we carried out an analysis based on the Essential Dynamics (ED) method^68^ (or Principal Component Analysis, PCA). More specifically, we use here a previously developed approach^69^ that extends the ED analysis, initially developed to analyze the collective motions of proteins, to investigate the collective fluctuation modes of a set of observables that contribute to the fluctuation of a functionally relevant target observable. Here, the set of observables that we address is given by the electrostatic potential of individual residues of the protein (${{{{\mathcal{V}}}}}_{{P}_{i}}$, also including, besides the protein amino acids, all chromophores and cofactors) along the MD trajectory and the target observable is the TE associated with the charge transfer between Chl_D1_ and Pheo_D1_. The goal is to identify a limited set of residues that are significant in determining the fluctuations of the TE.

**Fig. 6:**
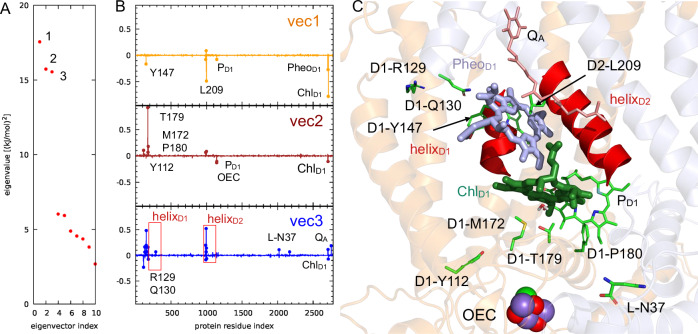
Principal component analysis of residue electrostatic potential fluctuations and their structural determinants of CT1 electron transfer modulation. **A** first ten eigenvalues of the covariance matrix of the single-residue electrostatic potentials as a function of the eigenvector index. **B** Components of the first three eigenvectors (vec 1, vec 2 and vec 3) of the single-residue potential covariance matrix. Dots indicate residues whose projection exceeds the 3*σ* threshold (0.02), highlighting those most responsible for collective electrostatic fluctuations. **C** Structural representation of the protein residues whose electrostatic potential fluctuations most strongly modulate the instantaneous electron transfer oscillations between ${{{{\rm{Chl}}}}}_{{{{\rm{D1}}}}}^{+}$ (dark green) and ${{{{\rm{Pheo}}}}}_{{{{\rm{D1}}}}}^{-}$ (lilac). The two helices closest to the CT pair  (residues 145-153 of the D1 chain and 197-212 of the D2 chain) are shown in red cartoon representation.

Panel A of Fig. 6 reports the first ten eigenvalues of the covariance matrix of the single-residue electrostatic potentials ${{{{\mathcal{V}}}}}_{{P}_{i}}$ as a function of the eigenvector index. As shown, the first three eigenvectors have much larger eigenvalues than the others, indicating dominant modes. We therefore analyzed the components of the corresponding eigenvectors (Fig. 6B) and extracted the residues with the highest contributions (the residues whose projection on an eigenvector exceeds 3*σ* (0.02) are marked with dots in Fig. 6B). The resulting subset of the protein consists of 27 residues, including both amino acids and chromophores. The significance of these residues in the instantaneous modulation of the TE is supported by the fact that the correlation between the sum of their potentials over the classical MD trajectory and the total electrostatic potential of the protein is 0.67 (see Suppl. Figure 5).

In the first eigenvector (vec1: yellow dots in Fig. 6), the CT pair (Chl_D1_ and Pheo_D1_) acts together with chlorophyll P_D1_, D1-Tyr147 (which interacts with the C=O of the phytol chain of Pheo_D1_), and D2-Leu209 (the closest residue to the Pheo_D1_ ring center). Moving to the second eigenvector (vec2, dark red dots), the largest projections are associated with residues facing the Chl_D1_ from the side where a water molecule is axially coordinated to its Mg ion. These residues include Met172, Thr179 and Pro180 from the D1 chain. Thr179, which shows the largest contribution, is particularly relevant due to its direct polar interaction with the Mg-coordinating water molecule. The relevance of such a close threonine residue has also been suggested previously^67^. A minor contribution from Tyr112 of the D1 chain is also observed. This residue forms a hydrogen bond with the backbone of Met172, making their coordinated motion plausible. A small contribution from P_D1_ and the OEC is also identified. It is noteworthy that the nearby and charged OEC residue, known to strongly influence the redox potential of the chromophores^26,70^, has only a minor role in the modulation of CT kinetics. Tyr147 and Leu209, which show relevant contributions in vec1, also contribute to eigenvector 3 (vec3: blue dots), along with several neighboring residues. All these residues belong to the two *α*-helices closest to the CT pair. Specifically, these helices are in the D1 chain (Val145-Ser153) and the D2 chain (Met198-Ala212) and are shown in red in Fig. 6C. Additional small contributions can be noted: D1-Gln130, which interacts with the C=O of the pheophytin ring; the nearby ionic residue D1-Arg129; the tail of quinone Q_*A*_, which lies very close to Pheo_D1_, and a distant ionic asparagine located 20 Å from Chl_D1_, corresponding to Asn37 in chain L of PDB:3WU2. Interestingly, some of the residues discussed above are also targets of point substitutions in genetic variants of the D1 protein^46^.

Taken together, the above results highlight that the instantaneous fluctuations of the TE are mainly driven by two regions of the proteins: that in between Chl_D1_ and the OEC cluster and that comprising the two above-described helices that define the protein pocket where the CT pair resides. In particular, the two helices are essential for modulating the TE. They act both through specific residue interactions, as highlighted in vec1, and via collective motion, as shown in vec3, which also involves apolar residues. The contribution of the two helices to the TE instantaneous fluctuations does not arise from a reorganization of the protein environment around the two CT partners (that cannot occur on such an ultrafast time scale) but rather from the variation of the CT partners/helices reciprocal orientation due to thermal fluctuations. Fluctuations in the relative distance and orientations between donor and acceptor due to thermal effects are also the origin of the contribution of the two CT partners to the TE instantaneous fluctuations. Importantly, we were able to determine these contributions in this work by treating the two CT partners as separate quantum centers (i.e., by performing separate QM calculations on SP and Chl_D1_, see Suppl. Note 2) rather than as a complex.

### Kinetic model

**Fig. 7:**
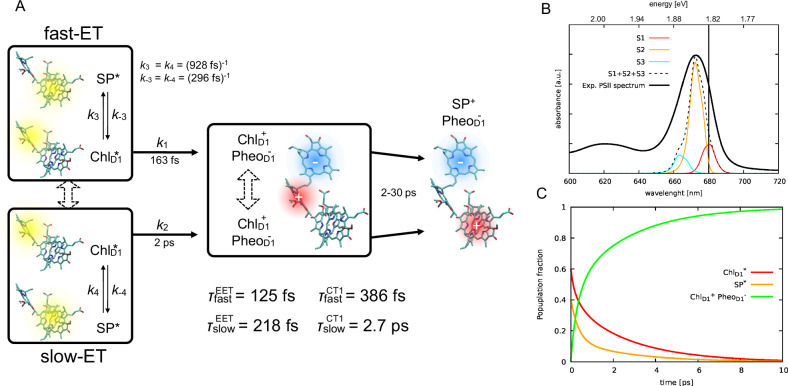
Kinetic model of charge separation in the PSII reaction center. **A** Kinetic scheme describing the processes involved in the primary and secondary charge-separation processes (electron transfer, ET, and excitation energy transfer, EET), from the initial photoexcitation in the reaction center to the formation of the metastable radical pair ${{{{\rm{SP}}}}}^{+}{{{{\rm{Pheo}}}}}_{{{{\rm{D1}}}}}^{-}$. **B** Experimental PSII absorption spectrum (black solid line, from ref. ^102^) compared with the PMM-calculated spectrum of the P_D1_P_D2_Chl_D1_ trimer (black dashed line). Details of the spectrum calculations are provided in the Methods section and in Suppl. Note 3. The vertical black line marks the pumping frequency at 680 nm. To align the peak positions, the energies in the calculated spectrum were scaled by a factor of 1.025. The PMM spectrum includes the first three excited states of the trimer the localization and CT character of which are reported in Suppl. Table 4. In particular, S1 is mostly localized on Chl_D1_ (Chl_D1_:75%;SP:25%); S2 is fully localized on SP, and S3 is mostly localized on SP with a component on Chl_D1_ (Chl_D1_:25%;SP:75%). **C** Time evolution of the relative populations of the key species involved in the excitation energy transfer and charge transfer processes obtained by solving the system of kinetic equations (see Suppl. Note 4): SP^*^ (orange), ${{{{\rm{Chl}}}}}_{{{{\rm{D1}}}}}^{*}$ (red), and the primary radical pair ${{{{\rm{Chl}}}}}_{{{{\rm{D1}}}}}^{+}{{{{\rm{Pheo}}}}}_{{{{\rm{D1}}}}}^{-}$ (green).

To compare our results with experimental literature data, we build a kinetic scheme and solve the corresponding differential equations to extract apparent lifetimes. Our kinetic scheme (see Fig. 7A) consists of the two parallel primary charge-separation pathways described above, each with compartments representing the starting excited states and the first radical pair (RP1). The pathways then converge to the ${{{{\rm{SP}}}}}^{+}{{{{\rm{Pheo}}}}}_{{{{\rm{D1}}}}}^{-}$ radical pair (RP2) by the transfer of an electron from the SP^+^ to ${{{{\rm{Chl}}}}}_{{{{\rm{D1}}}}}^{+}$ on a timescale of 2-30 ps, according to different experimental estimates. This step is not included in our model. Within the starting excited-state compartments, we consider for both water-modulated conformational states the internal electronic energy transfer between the primary electron donors SP^*^ and ${{{{\rm{Chl}}}}}_{{{{\rm{D1}}}}}^{*}$. We also consider the interconversion between the two conformational states. Late energy transfer from the two peripheral chlorophylls (Chlz_D1_, Chlz_D2_) is not included in the kinetic scheme.

To formulate the system of linear differential equations, the solution of which provides the complete kinetic relaxation (as described in Suppl. Note 4), one must define the seven microscopic rate constants indicated in Fig. 7A. The rate constants for the fast and slow primary CS paths are taken from the bi-exponential fit of the energy-variation upon electron transfer discussed above: *k*_1_ = (163 fs)^−1^ and *k*_2_ = (2.0 ps)^−1^, respectively. Concerning the EET processes, we use the rate constants obtained from the time-dependent populations of SP and Chl_D1_ provided by the quantum dynamics simulation, assuming the same values for both water-modulated conformational states. Finally, a rough estimate of the interconversion rate *k*_5_ (tens of picoseconds) can be obtained from the transition energy variation calculated over the 500 ps-long MD simulation of the ${{{{\rm{Chl}}}}}_{{{{\rm{D1}}}}}^{*}$ excited state. Note that, since this interconversion rate is much slower than *k*_1_ and *k*_2_, its exact value is not critical and does not influence the final, observed time constants.

Regarding the choice of the initial population of the different states, we aim to model the kinetics following the direct excitation of the primary donors, i.e., the special pair and the accessory chlorophyll Chl_D1_ at 680 nm (on the red side of the main peak), as done in many experiments ^11,14,15^. In our model the excited-state manifold includes the excited states of the SP and Chl_D1_ in both water-modulated conformational states of the system. To reproduce the experimental pump, the initial populations are determined from the delocalization of the first two excited states, as obtained from participation ratio (PR) analysis, and from the corresponding absorption intensity on the red side of the main peak, calculated within the MD-PMM approach (see Fig. 7B) providing 49% ${{{{\rm{Chl}}}}}_{{{{\rm{D1}}}}}^{*}$ (red) and 51% SP^*^(orange); for the details of the calculations of the absorption spectrum and of the participation ratio to the first three excited states see Suppl. Note 3. These were then weighted with the relative populations of the two conformational states (namely 40% for the slow-ET regime and 60% for fast-ET regime, see Fig. 3). The resulting starting populations are: P(${{{{\rm{Chl}}}}}_{{{{\rm{D1}}}}}^{*}$, fast-ET) = 29%, P(${{{{\rm{Chl}}}}}_{{{{\rm{D1}}}}}^{*}$, slow-ET) = 20%, P(SP^*^, fast-ET) = 31% and P(SP^*^, slow-ET) = 20%. It should be noted that the absorption spectrum calculated in the present case includes only three out of at least eight chromophores typically absorbing at 680 nm in isolated RC complexes (four core chlorophylls a, two peripheral chlorophylls a, and two pheophytins). The fact that our model neglects contributions from the remaining pigments, which are known to broaden the spectral distribution through excitonic interactions, and that quantum vibrational effects are not included, is expected to lead to an underestimation of the width. However, our aim here is not to perform a detailed comparison with experiment, as was successfully done, for example, for the CP43 complex^71^ which contains 14 chlorophylls, but rather to establish the relative intensities of Chl_D1_ and SP at 680 nm for defining the starting populations in the kinetic model.

Because the interconversion rate between the two conformational basins *k*_5_ (Fig. 7A) is much slower than all intra-basin rates, its exact value does not influence the apparent time constants (see Suppl. Note 4), and the kinetics of the two basins can be treated analytically as two kinetically independent three-level systems. Analytical diagonalization of the corresponding rate matrices yields the observable time constants directly from the eigenvalues of the coupled kinetic scheme (see Fig. 7A). Figure 7C displays the time evolution of the ${{{{\rm{Chl}}}}}_{{{{\rm{D1}}}}}^{+}{{{{\rm{Pheo}}}}}_{{{{\rm{D1}}}}}^{-}$ radical pair population (green), the total ${{{{\rm{Chl}}}}}_{{{{\rm{D1}}}}}^{*}$ population (as the sum of P(${{{{\rm{Chl}}}}}_{{{{\rm{D1}}}}}^{*}$, fast-ET) and P(${{{{\rm{Chl}}}}}_{{{{\rm{D1}}}}}^{*}$, slow-ET), red), and the total SP^*^ population (as the sum of P(SP^*^, fast-ET) and P(SP^*^, slow-ET), orange). All state populations are shown in Suppl. Figure 6.

The model identifies four distinct time constants: 125 fs, 218 fs, 386 fs and 2.7 ps. Inspection of the eigenvectors reveals that in both basins the faster eigenmode exhibits an anti-phase relationship between the SP^*^ and ${{{{\rm{Chl}}}}}_{{{{\rm{D1}}}}}^{*}$ components, characteristic of EET pre-equilibration, while the slower eigenmode shows in-phase co-decay of both excited states into the radical pair, characteristic of CT1. Thus, the sub-250 fs components make a negligible contribution to radical pair formation. The four apparent time constants are therefore naturally assigned as: ${\tau }_{{{{\rm{fast}}}}}^{{{{\rm{EET}}}}}=125$ fs (EET, fast basin), ${\tau }_{{{{\rm{slow}}}}}^{{{{\rm{EET}}}}}=218$ fs (EET, slow basin), ${\tau }_{{{{\rm{fast}}}}}^{{{{\rm{CT1}}}}}=386$ fs (CT1, fast basin), and ${\tau }_{{{{\rm{slow}}}}}^{{{{\rm{CT1}}}}}=2.7$ ps (CT1, slow basin).

We recognize that a detailed comparison of the computed apparent rate constants with experimental results is challenging due to intrinsic differences such as fitting models, sample preparation, and experimental parameters. Nevertheless, the computed results are generally consistent with experimental observations, as shown in Table 1, which compares the apparent time constants obtained in this work with experimental data, giving confidence to the theoretical predictions.

**Table 1:** Comparison between the apparent time constants obtained in the present work and experimental values reported for the isolated PSII reaction center, measured up to ≈ 5 ps and assigned up to the primary charge-transfer step

	This work	Klug^73^	Van Grondelle^19^	Rögner^11^	Renger^21^	Ogilvie^72^	Van Grondelle^15^	Shuvalov^16^	Miller^17^	Fleming^22^	Ogilvie^18^
< 0.25 ps	0.12 (EET)	0.1 (EET)	–	0.1–0.2 (NA)	–	0.05–0.15 (EET)	0.2 (CT_*SP*_)	–	0.13 (EET+CT_*SP*_)	–	–
	0.22 (EET)										
0.25–1 ps	0.39 (CT1)		0.6–0.8 (CT1)	0.6 (EET)	0.3 (CT1)	–	0.7 (CT1)	0.9 (CT3)	–	–	0.45 (CT1)
1–5 ps	2.7 (CT1)	3.0 (CT1)	–	3.2 (CT1)	–	1–3 (CT1)	3.0 (CT3)	–	1.5 (CT1)	1.3 (CT1+EET)	

The assignments reported in the respective studies are indicated in parentheses in agreement with the nomenclature of Fig. 1 (CT-SP: ${{{{\rm{P}}}}}_{{{{\rm{D1}}}}}^{-}{{{{\rm{P}}}}}_{{{{\rm{D2}}}}}^{+}$; CT1: ${{{{\rm{Chl}}}}}_{{{{\rm{D1}}}}}^{+}{{{{\rm{Pheo}}}}}_{{{{\rm{D1}}}}}^{-}$; CT3: ${{{{\rm{P}}}}}_{{{{\rm{D1}}}}}^{+}{{{{\rm{Chl}}}}}_{{{{\rm{D1}}}}}^{-}$; NA = not assigned). All time constants are given in picoseconds. Measurements in Refs. ^11,17,19,21,73^ were performed at room temperature, whereas those in Refs. ^14–16,18,22,72^ were carried out at cryogenic temperature.

The two fastest (<250 fs) calculated time constants (125 fs and 218 fs), associated with the EET process occurring within the reaction center, are in good agreement with several time constants reported in the literature based on experimental data and attributed to EET processes occurring within the reaction center^72,73^, and could be compatible with apparent time constants that were attributed either to the ${{{{\rm{P}}}}}_{{{{\rm{D1}}}}}^{-}{{{{\rm{P}}}}}_{{{{\rm{D2}}}}}^{+}$ formation^15^, or to a mixed EET/${{{{\rm{P}}}}}_{{{{\rm{D1}}}}}^{-}{{{{\rm{P}}}}}_{{{{\rm{D2}}}}}^{+}$ process^17^. The calculated ${\tau }_{{{{\rm{fast}}}}}^{{{{\rm{CT1}}}}}=$386 fs, corresponding to charge-separation dynamics, is consistent with time constants in the range of 300–900 fs, which are mainly attributed in the literature to the primary charge-separation steps^15,16,18,19,21^. ${\tau }_{{{{\rm{slow}}}}}^{{{{\rm{CT1}}}}}=$2.7 ps, also associated with charge separation, is in excellent agreement with the time constants consistently attributed to the primary CT in the literature, which range from 1 to 5 ps^11,15,17,22,72^. Moreover, ${\tau }_{{{{\rm{fast}}}}}^{{{{\rm{CT1}}}}}$ and ${\tau }_{{{{\rm{slow}}}}}^{{{{\rm{CT1}}}}}$ agree with the two constants reported by Novoderezhkin et al. (700 fs and 3 ps)^15^, which were attributed to the two different ${{{{\rm{Chl}}}}}_{{{{\rm{D1}}}}}^{+}$ and ${{{{\rm{Chl}}}}}_{{{{\rm{D1}}}}}^{-}$ pathways. Here, we instead attribute them to two parallel ${\,{{{\rm{Chl}}}}}_{{{{\rm{D1}}}}}^{+}$ pathways occurring within the two ET regimes that are modulated by solvent organization. The present scheme reconciles previously reported, distinct estimates of the CT1 lifetime, some on the order of hundreds of femtoseconds^15,18,19,21^ and others on the picosecond timescale^11,17,22,72^.

## Discussion

Understanding the kinetics of charge separation in Photosystem II is a long-standing challenge in photosynthesis research. A major source of uncertainty stems from the similar timescales of energy transfer and charge separation, along with the extensive spectral overlap of pigment transitions in the Q_*y*_ region. These complexities have led to conflicting hypotheses regarding the number and identity of primary charge-separated states and their formation sequence.

Here, we present a model for the primary charge-separation kinetics in the 100 fs to a few picoseconds range. Previously, the observed bi-exponential behavior was attributed to parallel pathways involving different intermediate radical pairs^14,15^, but our analysis offers a different perspective. We propose that the ${\,{{{\rm{Chl}}}}}_{{{{\rm{D1}}}}}^{+}$ pathway, i.e., the direct reduction of Pheo_D1_ by the excited Chl_D1_, is the dominant charge-separation route, and that the bi-exponential kinetics instead reflect conformational heterogeneity within this pathway. This conclusion is supported by a detailed examination of how the energy difference associated with primary charge-separation correlates with motions of protein and solvent molecules around the exchanging chromophores. Our analyses show that the interconversion between the fast (hundreds of femtoseconds) and slower (a few picoseconds) regimes is driven by dynamic rearrangements of water molecules within the protein matrix, including the water channels that regulate access to the oxygen-evolving complex. These findings clarify the molecular basis of the hierarchical timescales in primary charge transfer, highlighting the distinct and complementary roles of both protein and solvent dynamics in shaping electron transfer kinetics.

In addition to rationalizing the bi-exponential behavior of the primary charge separation, we also investigate the excitation energy-transfer redistribution within the RC and find that it occurs on a timescale of around 200 fs. Putting together the above results, we propose a kinetic model based on two parallel charge-separation pathways arising from the bi-exponentiality of the primary charge separation. By solving the corresponding differential equations we extract apparent lifetimes to be compared with experimental results. The apparent rate constants obtained from solving the full kinetic model are 125-218 fs, 386 fs and 2.7 ps. These three timescales agree well with the distinct experimentally measured time regimes. The theoretical model provides molecular-scale insight into the mechanisms underpinning these dynamics, enabling us to identify the dominant charge-separation pathway and elucidate the molecular basis of distinct kinetic components, despite the inherent complexity of this system.

## Methods

### Quantum dynamics of energy transfer

The energy-transfer dynamics within the P_D1_ − P_D2_ − Chl_D1_ triad are simulated using a Frenkel-Holstein Hamiltonian, which couples electronic site energies and inter-site excitonic couplings to a vibrational bath. The bath spectral density combines a Drude term for low-frequency protein fluctuations (*λ*_*D*_ = 35 cm^−1^, Ω_*D*_ = 40 cm^−1^) with 48 under-damped intramolecular modes parameterized from experiment, discretized into 150 bath modes per site. Finite-temperature effects are incorporated via thermo-field dynamics (TFD) through a thermal Bogoliubov transformation. The time evolution of the full system-bath wavefunction is propagated using the time-dependent density matrix renormalization group (TD-DMRG) algorithm, in which the wavefunction is represented as a matrix product state (MPS) with virtual bond dimension *M* = 50, a simple harmonic oscillator basis of *d* = 30, and a time step of Δ*t* = 1 fs. Propagation employs the time-dependent variational principle (TDVP) with a second-order projector-splitting scheme, as implemented in RENORMALIZER^74^.

### The MD-PMM approach

The MD-PMM approach^40^, like other hybrid quantum/classical methods^75–78^, divides the system under study into two regions: (1) the quantum center (QC), which includes the atoms involved in the processes of interest (such as charge transfer reactions) and is treated quantum mechanically, and (2) the remainder of the system (the environment), which influences the QC’s properties through an electrostatic perturbation derived from the system’s classical MD configurations. A key advantage of the MD-PMM is that it allows the perturbed properties of the QC to be evaluated using numerous atomistic configurations of the environment, which are obtained from extended classical MD simulations of the entire system (including the quantum part). In the MD-PMM, the perturbation operator is expanded so that the effect of the environment is applied a posteriori to the quantum properties of the QC. At first, the quantum properties of the QC (i.e., the unperturbed properties) are calculated using quantum-mechanical methods in the gas phase. Then, for each configuration/frame generated by the all-atoms classical MD simulations, the QC Hamiltonian operator is perturbed by the instantaneous electrostatic field resulting from the environment’s configuration. Thus, the electronic Hamiltonian operator ${\widehat{H}}_{k}$ of the QC in the presence of the perturbing environment in configuration *k* is:

5 $${\widehat{H}}_{k}={\widehat{H}}^{0}+{\widehat{V}}_{k}$$

Here, ${\widehat{H}}^{0}$ is the unperturbed Hamiltonian of the QC, computed considering the isolated QC, and ${\widehat{V}}_{k}$ is the perturbation operator derived from the environment’s configuration *k*:

6 $${\widehat{V}}_{k}\approx {q}_{T}{{{{\mathcal{V}}}}}_{k}({{{{\bf{r}}}}}_{0})-{{{{\bf{E}}}}}_{k}({{{{\bf{r}}}}}_{0})\cdot \widehat{{{{\boldsymbol{\mu }}}}}$$

where *q*_*T*_, **r**_**0**_, and $\widehat{{{{\boldsymbol{\mu }}}}}$ are the QC’s total charge, center of mass, and dipole operator, respectively; ${{{{\mathcal{V}}}}}_{k}$ is the electrostatic potential exerted by the perturbing environment’s configuration *k* and **E**_*k*_ is the corresponding perturbing electric field (${{{\bf{E}}}}=-\partial {{{\mathcal{V}}}}/\partial {{{\bf{r}}}}$).

At each frame of the MD simulation ensemble (i.e., for each environment configuration *k*), the perturbed electronic Hamiltonian matrix is constructed and diagonalized, providing a continuous trajectory of perturbed eigenvalues and eigenvectors. The instantaneous perturbed quantum observable of interest for the QC can then be evaluated for each configuration of the environment.

For the calculation of the transition energy associated with the CT1 reaction (see also Suppl. Note 2), the unperturbed (gas-phase) quantum-mechanical properties of each chromophore are computed individually - the chlorophyll and pheophytin are treated as separate quantum centers rather than as a single quantum system. Initial geometries are obtained through QM/MM geometry optimization with electrostatic embedding, as implemented in ORCA^79^. The energies, dipole moments, and vertical excitation energies of the isolated chromophores are then calculated using time-dependent density functional theory (TD-DFT)^80^, employing the *ω*B97X-V functional^81,82^ and the Def2-TZVP basis set^83,84^. These properties are computed for the donor chromophore in both its excited and oxidized states, and for the acceptor in its neutral and reduced states.

The environment perturbation (Eq. (6)) is then applied to both the excited and oxidized states of the donor (Chl_D1_) and to the neutral and reduced states of the acceptor (Pheo_D1_). Diagonalization of the perturbed Hamiltonian (Eq. (5)) provides perturbed energies for the donor in its excited and oxidized states (${\epsilon }_{{{{{\rm{Chl}}}}}_{{{{\rm{D}}}}1}^{*}}$ and ${\epsilon }_{{{{{\rm{Chl}}}}}_{{{{\rm{D}}}}1}^{+}}$) and for the acceptor in its neutral and reduced states (${\epsilon }_{{{{{\rm{Pheo}}}}}_{{{{\rm{D1}}}}}}$ and ${\epsilon }_{{{{{\rm{Pheo}}}}}_{{{{\rm{D}}}}1}^{-}}$). The transition energy (TE) for each *k*-th MD configuration is then calculated as the energy difference between the perturbed energies of the donor/acceptor pair in the product state and in the reactant state (Eq. (4)). Within this semi-classical simulation framework, no actual charge transfer occurs during the simulation. Rather, the condition necessary for charge separation is identified as the instant at which TE equals zero (sudden degeneracy between reactant and product states). This estimate is corrected by applying an adiabaticity factor computed using the Landau-Zener approximation^85^.

The UV-Vis absorption spectrum is computed within the PMM framework (see also Suppl. Note 3) using the same MD trajectory and perturbation protocol described above. The quantum properties of the excitable trimer composed of P_D1_P_D2_Chl_D1_ are calculated as a single quantum system using TD-DFT at the same level of theory described above (*ω*B97X-V/Def2-TZVP), including the first three excited states. The unperturbed properties of this trimer are subsequently perturbed using configurations drawn from the 200 ns ground-state classical MD trajectory. For each perturbed excited state, the distribution of transition frequencies is accumulated with an appropriate number of bins in frequency space to yield a smooth and detailed curve. The molar extinction coefficient *ε*_0,*i*_ for the ground-to-*i*th excited-state transition is then obtained from these distributions within the vertical transition approximation:

7 $${\varepsilon }_{0,i}(\nu )\cong {\sum}_{{{{\rm{\nu }}}}_{{{{\rm{ref}}}}}}\frac{{\Gamma }_{A}({{{\rm{\nu }}}}_{{{{\rm{ref}}}}})\,n({{{\rm{\nu }}}}_{{{{\rm{ref}}}}})\,h{{{\rm{\nu }}}}}{N}\frac{{e}^{-\frac{{({{{\rm{\nu }}}} -{{{\rm{\nu }}}}_{{{{\rm{ref}}}}})}^{2}}{2{\sigma }^{2}}}}{\sigma \sqrt{2\pi }}$$

8 $${\Gamma }_{A}({{{\rm{\nu }}}}_{{{{\rm{ref}}}}})=\frac{| {{{{\boldsymbol{\mu }}}}}_{0,i}{| }_{{{{\rm{\nu }}}}_{{{{\rm{ref}}}}}}^{2}}{6{\epsilon }_{0}\,c^{2}}$$

where *ν*_ref_ is the frequency at the center of each bin, *n*(*ν*_ref_) is the corresponding number of MD frames, and $| {{{{\boldsymbol{\mu }}}}}_{0,i}{| }_{{\nu }_{{{{\rm{ref}}}}}}^{2}$ is the squared norm of the transition dipole moment. *ℏ* = *h*/(2*π*) with *h* the Planck constant, *ϵ*_0_ is the vacuum permittivity, *c* is the speed of light, and *σ*^2^ is the variance accounting for semi-classical vibrational contributions around each reference structure. A multiplicative energy scaling factor of 1.025 was applied to align the calculated absorption maximum with the experimental peak at approximately 672 nm.

### Classical MD simulations

The 1.9 Å resolution crystal structure of Photosystem II (3WU2) was used as the basis for constructing the molecular mechanics model^86^. The PSII monomer was embedded within a POPC (1-palmitoyl-2-oleoyl- sn-glycero-3-phosphocholine) lipid bilayer (a combined total of 784 POPC lipid molecules were added in the upper and lower leaflet), and water molecules were added to the stromal and lumenal sides (along the *z*-axis, perpendicular to the membrane plane). Na^+^ and Cl^−^ counterions were included to achieve a physiological salt concentration of 0.15 M. The final system dimensions were 176 Å × 176 Å × 160 Å, comprising a total of 512,341 atoms. The bonded parameters of all organic cofactors were based on the General AMBER Force Field(GAFF2)^87^. The bonded parameters for the non-heme iron site^88^, chlorophyll *a*^89^ and heme^90^ sites were taken from the literature. On the other hand, custom bonded parameters^66^ were used for the oxygen-evolving cluster, Mn_4_CaO_5_. The parameters for the protein residues, water and lipid bilayer were defined based on the Amber14SB^91^, TIP3P^92^ and LIPID14 force-fields^93^, respectively. The electrostatic charges of all cofactors were based on the Merz-Kollman Restrained Electrostatic Potential (MK-RESP) strategy^94^. All simulations were conducted using the AMBER18 molecular dynamics package^95,96^. The SHAKE algorithm^97^ was applied to constrain bonds involving hydrogen atoms, enabling a time step of 2 fs. Long-range electrostatic interactions were handled using the Particle Mesh Ewald (PME) method^98^ with a 1.0 nm cut-off. The temperature of the MD was set at 303 K and controlled during the simulations using Langevin dynamics^99^, with a collision frequency of 5 ps^−1^ during heating steps and 1 ps^−1^ during stable temperature phases. Pressure was regulated anisotropically using the Berendsen barostat^100^ with a relaxation time of 2 ps and a target pressure of 1 bar. The isothermal compressibility was set to 4.5 ⋅ 10^−5^bar^−1^. The ground-state MD production trajectory was previously performed^26,29^ and consists of 200 ns saved every 10 ps. The ${{{{\rm{Chl}}}}}_{{{{\rm{D1}}}}}^{*}$ MD used for the transition energy calculations was started from the end of the ground-state trajectory and extended for 500 ps saved every 20 fs. In this case, only the partial charges of Chl_D1_ of the original force-field were substituted with the excited ones. Such charges were calculated with the Restrained Electrostatic Potential fitting implemented in Gaussian16^101^ and using the same level of theory described above and in Suppl. Note 2.

## Supplementary information

Supplementary Information

Description of Additional Supplementary File

Supplementary Data 1

Supplementary Data 2

Transparent Peer Review file

## Source data

Source Data 1

Source Data 2

## Data Availability

All data supporting the findings of this study are available within the paper and its Supplementary Information file. The input files, force-field parameters, and starting and final structures of the classical MD simulations are provided as Supplementary Data 1. The 11 QM/MM optimized structures of the trimer are provided as Supplementary Data 2. All data supporting the figures in the main manuscript are provided as Source Data 1. All data supporting the Supplementary Figs. are provided as Source Data 2. A detailed description of all supplementary datasets and files is available in the document entitled “Description of Additional Supplementary Files”. Source data are provided with this paper.
