# Supplementary material for: Water-modulated conformational heterogeneity underlies multiple timescales of primary charge separation in photosystem II
Source: Nat Commun. 2026 Jun 20;17:7795. doi: 10.1038/s41467-026-73961-w (PMC13439440; doi:10.1038/s41467-026-73961-w)
Supplement: Supplementary file 1 [file 41467_2026_73961_MOESM1_ESM.pdf]

Water-Modulated Conformational Heterogeneity Underlies Multiple Timescales of Primary Charge Separation in Photosystem II

Matteo Capone[1], Alfy Benny[2], Gianluca Dell'Orletta[3], Gregory D. Scholes[2,*], Dimitrios A. Pantazis[4,*], Laura Zanetti-Polzi[1,*], Isabella Daidone[3,*]

June 19, 2026

[1] Institute of Nanoscience - CNR, S3, Via G. Campi 213/A, Modena, Italy

[2] Department of Chemistry, Frick Laboratory, Princeton University, Princeton, New Jersey 08544, United States

[3] Department of Physical and Chemical Sciences, University of L'Aquila, via Vetoio (Coppito 1), 67010 L'Aquila, Italy

[4] Max-Planck-Institut für Kohlenforschung, Kaiser-Wilhelm-Platz 1, 45470 Mülheim an der Ruhr, Germany

[*] Corresponding authors: gscholes@princeton.edu, dimitrios.pantazis@kofo.mpg.de, laura.zanettipolzi@nano.cnr.it, isabella.daidone@univaq.it

Contents

Suppl. Note 1. Quantum Dynamics 3

Suppl. Note 1.1. Electronic couplings 5

Suppl. Note 2. Transition Energy (TE) calculation 7

Suppl. Note 3. UV-Vis spectra calculation 8

Suppl. Note 3.1. Excited state analysis 8

Suppl. Note 4. Kinetic Model of the Reaction Scheme 11

List of Figures

Suppl. Figure 1. Time evolution of the transition energy (TE, panel A) and electrostatic potential contribution to the transition energy ($q\mathcal{V}$, panel B) without the inclusion of the contribution of the counterions. In panel C, the correlation between the quantities is shown. Both TE and $q\mathcal{V}$ are normalized to be dimensionless, with zero mean and unit variance. 13

Suppl. Figure 2. Correlation between the instantaneous and $q\mathcal{V}$ fluctuations with the fluctuations of $q\mathcal{V}_p$ and $q\mathcal{V}_s$, respectively. A: time evolution of $q\mathcal{V}$; B: time evolution of $q\mathcal{V}$ in which fast fluctuations are filtered by performing a rolling average; C: time evolution of $q\mathcal{V}$ in which slow fluctuations are filtered (obtained by subtracting the trend in panel B from that in panel A). D: time evolution of $q\mathcal{V}_s$ in which fast fluctuations are filtered by performing a rolling average; E: time evolution of $q\mathcal{V}_p$. F: correlation between $q\mathcal{V}_s$ and $q\mathcal{V}$ both with fast fluctuations filtered ($R \approx$0.8). G: correlation between $q\mathcal{V}_p$ and $q\mathcal{V}$ with slow fluctuations filtered ($R \approx$0.8). All quantities are normalized to be dimensionless, with zero mean and unit variance. 14

Suppl. Figure 3. Correlation between $q\mathcal{V}_s$ and the electrostatic potential exerted by the internal water molecules (i.e., water molecules within 25 Å of $\mathrm{Chl_{D1}}$ and $\mathrm{Pheo_{D1}}$ and belonging to the OEC channels) $q\mathcal{V}_{int}$. Both quantities are normalized to be dimensionless, with zero mean and unit variance. 15

Suppl. Figure 4. Time evolution of TE and the electrostatic potential exerted by the water molecules belonging to the Cl1 channel ($q\mathcal{V}_{\mathrm{Cl1}}$). Both quantities are normalized to be dimensionless, with zero mean and unit variance. 16

Suppl. Figure 5. Left panels: time evolution of the protein electrostatic potential (top) and sum of the protein electrostatic potential of selected residues from the first three eigenvectors calculated with ED analysis (bottom). Right panel: correlation between $q\mathcal{V}$ of the protein and $q\mathcal{V}$ of the residue selection. Both quantities are normalized to be dimensionless, with zero mean and unit variance. 17

Suppl. Figure 6. Time evolution of the relative populations of the key species involved in excitation energy transfer and charge transfer processes obtained by solving the system of kinetic equations. The species are: $\mathrm{Chl^{*}_{D1}}$ in a fast (violet) and slow (magenta) regime; SP* in a fast (brown) and slow (orange) regime; and the radical pair $\mathrm{Chl^{+}_{D1}Pheo^{-}_{D1}}$ (green). 18

Suppl. Note 1 Quantum Dynamics

The energy transfer in the P_{D1}-P_{D2}-Chl_{D1} triad system is studied based on a Frenkel-Holstein model:

$$H = \sum_i E_i a_i^\dagger a_i + \sum_{ij} V_{ij} a_i^\dagger a_j + \sum_{in} \omega_{in} b_{in}^\dagger b_{in} + \sum_{in} g_{in} a_i^\dagger a_i (b_{in}^\dagger + b_{in}) \tag{1}$$

Where E_i is the site energies with inter-site excitonic coupling V_{ij}. We have used $E_{\mathrm{PD1}} = 14720$ cm^{-1}, $E_{\mathrm{PD2}} = 14650$ cm^{-1}, and $E_{\mathrm{ChlD1}} = 14460$ cm^{-1}; $V = 150$ cm^{-1}, J_1 = -42 cm^{-1} and J_2 = -56 cm^{-1} for P_{D1}-P_{D2}, P_{D1}-Chl_{D1} and P_{D2}-Chl_{D1} pairs, respectively, to be consistent with previous literature [1, 2, 3] (see section Suppl. Note 1.1). The bath frequencies (ω_{in}) and electron-photon coupling strengths (g_{in}) are obtained by discretizing the spectral density ($J(\omega)$) (*vide infra*). In the present work, $n = 150$ discrete modes are used, sampled uniformly from $J(\omega)$.

The bath coupling factors g_n are obtained from an effective spectral density consisting of low-frequency protein effects and high-frequency intramolecular vibrations [1]. The low-frequency region is modeled by a Drude spectral density as follows:

$$J_D(\omega) = \frac{2\lambda_D \omega \Omega_D}{(\omega^2 + \Omega^2)} \tag{2}$$

with $\lambda_D = 35$ cm^{-1} and $\Omega_D = 40$ cm^{-1}. For the intramolecular effects, we use the experimentally measured spectral density modeled using 48 under-damped modes (Table S1) to get $J(\omega) = \sum_i G_i(\omega)$, where:

$$G_i(\omega) = \frac{2\lambda_i \omega_i^2 \gamma_i \omega}{(\omega^2 - \omega_i^2)^2 + \gamma_i^2 \omega^2} \tag{3}$$

Hence, the total spectral density is taken as $J(\omega) = J_D(\omega) + \sum_i G_i(\omega)$

ω_j	s_j	ω_j	s_j	ω_j	s_j	ω_j	s_j
97	0.0371	604	0.0034	1143	0.0303	1354	0.0057
138	0.0455	700	0.0050	1181	0.0179	1382	0.0067
213	0.0606	722	0.0074	1190	0.0084	1439	0.0067
260	0.0539	742	0.0269	1208	0.0121	1487	0.0074
298	0.0488	752	0.0219	1216	0.0111	1524	0.0067
342	0.0438	795	0.0077	1235	0.0034	1537	0.0222
388	0.0202	916	0.0286	1252	0.0051	1553	0.0091
425	0.0168	986	0.0162	1260	0.0064	1573	0.0044
518	0.0303	995	0.0293	1286	0.0047	1580	0.0044
546	0.0030	1052	0.0131	1304	0.0057	1612	0.0044
573	0.0094	1069	0.0064	1322	0.0202	1645	0.0034
585	0.0034	1110	0.0192	1338	0.0037	1673	0.0010

Suppl. Table 1: Vibrational frequencies ω_j (cm^{-1}) and Huang–Rhys factors s_j taken from Ref. [1].

The finite-temperature effects are incorporated using thermo-field dynamics (TFD) via the thermal Bogoliubov transformation [4, 5]. The resulting system bath Hamiltonian is:

$$\bar{H}_{SB} = \sum_{in} \omega_{in}(b_{in}^\dagger b_{in} - \tilde{b}_{in}^\dagger \tilde{b}_{in}) + \sum_{in} g_{in} a_i^\dagger a_i \left[\cosh(\theta_{in})(b_{in}^\dagger + b_{in}) + \sinh(\theta_{in})(\tilde{b}_{in}^\dagger + \tilde{b}_{in})\right] \tag{4}$$

where $\tilde{b}_{in}$ are the auxiliary bosonic operators and $\theta_n = \mathrm{arctanh}(e^{-\beta\omega_n/2})$. Hence, with 3 sites, we use 450 overall vibrational DoF, which is effectively doubled due to the TFD Hamiltonian.

The coherence between sites are calculated as the sum of the absolute values of the off-diagonal elements of the reduced density matrix as $C(i,j) = \sum_{i\neq j} |\rho_{ij}|$, where i and j are indices corresponding to the P_{D1}, P_{D2} and Chl_{D1} sites.

The quantum dynamics is simulated using time dependent density matrix renormalization group (TD-DMRG) algorithm. Here, the wavefunctions $|\psi\rangle$ are represented as matrix product states (MPS):

$$|\Psi\rangle = \sum_{\{\sigma\}} C_{\sigma_1\sigma_2\ldots\sigma_M} |\sigma_1\sigma_2\ldots\sigma_N\rangle = \sum_{\{a\},\{\sigma\}} A[1]^{\sigma_1}_{a_1} A[2]^{\sigma_2}_{a_1,a_2} \ldots A[N]^{\sigma_N}_{a_{N-1}} |\sigma_1\sigma_2\ldots\sigma_N\rangle \tag{5}$$

The $A[n]^{\sigma_n}_{a_{n-1}a_n}$ refers to the local rank 3 tensor for the nth DoF. $|\sigma_n\rangle$ is the basis set of each DoF, and the dimension of σ_n of the rank 3 tenor represents the physical bond dimension d. The dimension of a_n corresponds to the virtual bond dimension M. To be compatible with MPS, the operators $(\hat{O})$ is expressed in the matrix product operator (MPO) form:

$$\hat{O} = \sum_{\{w\},\{\sigma\},\{\sigma'\}} W[1]^{\sigma'_1,\sigma_1}_{w_1} W[2]^{\sigma'_2\sigma_2}_{w_1w_2} \cdots W[N]^{\sigma'_N,\sigma_N}_{w_{N-1}} \left|\sigma'_1\sigma'_2\cdots\sigma'_N\right\rangle \langle\sigma_N\sigma_{N-1}\cdots\sigma_1| \tag{6}$$

A simple harmonic oscillator (SHO) basis of $d = 30$ is used, with virtual bond dimension $M = 50$ and time propagation is performed with a time step of $\Delta t = 1$ fs. The time dependent variational principle (TDVP) is used to propagate the initial wavefunction using TD-DMRG formalism.

$$\frac{\partial|\Psi(t)\rangle}{\partial t} = -i\hat{P}\hat{H}|\Psi(t)\rangle \tag{7}$$

where $\hat{P}$ represents the tangent space projector operator determined by $|\Psi(t)\rangle$. By substituting Eq. 5 and Eq. 6 to Eq. 7, we can derive equations of motions for all coefficients $A[n]^{\sigma_n}_{a_{n-1}a_n}$ of an MPS. The second order projector-splitting scheme is employed for higher efficiency propagation. More information could be found at Ref. [4]. The TD-DMRG dynamics is carried out using RENORMALIZER [6].

Suppl. Note 1.1 Electronic couplings

The quantum dynamics treatment of the energy transfer based on the TD-DMRG algorithm requires the use of inter-site couplings for the P_{D1}-P_{D2}-Chl_{D1} triad system. This trimer system is oriented such that the P_{D1} and P_{D2} are strongly coupled: their spatial proximity gives rise to short-range effects including coupling between local and charge transfer (CT) excited states. The inclusion of short-range effects within the SP was pursued by Renger and coworkers both by using multiscale simulations and a diabatization scheme combining the Fragment Charge Difference (FCD) [7] and the Fragment Excitation Difference (FED) [8, 9] methods [10] and by fitting experimental data [2]. The value of the electronic coupling V between P_{D1} and P_{D2} used in our quantum dynamics simulations (V= 150 cm^{-1}) was obtained from fits of optical spectra [2], empirically incorporating the environmental and short-range effects. The coupling values J_1 = -42 cm^{-1} and J_2 = -56 cm^{-1} used in our quantum simulations for the P_{D1}-Chl_{D1} and P_{D2}-Chl_{D1} pairs, respectively, were obtained using the Transition Electrostatic Potential (TrESP) approach with a rescaling procedure to account for environmental effects. To assess the possible relevance of short range effects on the J_1 and J_2 couplings, we compute the full couplings of the individual P_{D1}-Chl_{D1} and P_{D2}-Chl_{D1} pairs using a diabatization approach and compare them to those obtained with the TrESP approach.

Couplings were computed for the individual pigment-pigment pairs P_{D1}-Chl_{D1} and P_{D2}-Chl_{D1} using the Fragment Excitation Difference (FED) method [8, 9] as implemented in the Q-Chem 5.4.2 package [11]. Considering a dimer composed by two fragments A and B (in the present case, Chl_{D1} and either P_{D1} or P_{D2}), the FED approach employs a diabatization scheme to recover the localized basis from delocalized excited states by means of the excitation difference matrix Δx with elements:

$$\Delta x_{mn} = x^B_{mn} - x^A_{mn} = \int_{r\ \in \mathrm{B}} \rho^{(mn)}_{ex}(\mathbf{r})d\mathbf{r} - \int_{r\ \in \mathrm{A}} \rho^{(mn)}_{ex}(\mathbf{r})d\mathbf{r} \tag{8}$$

where $\rho^{(mn)}_{\mathrm{ex}}(\mathbf{r})$ is the sum of attachment and detachment densities for transition $|m\rangle \rightarrow |n\rangle$.

The choice of applying the FED approach, which is valid for purely excitonic excited states, to the dimers composed of P_{D1}-Chl_{D1} and P_{D2}-Chl_{D1} is justified by the excited-state analysis (section Suppl. Note 3.1) which showed that no charge-transfer character is present between the special pair and Chl_{D1} in the first 5 excited states. The same does not hold true for the P_{D1}-P_{D2} coupling, for which charge-transfer excited states have been shown to contribute significantly to the coupling magnitude. An accurate evaluation of this coupling would therefore require a more refined approach, as addressed in Ref. [10]. Since a rigorous calculation of the P_{D1}-P_{D2} coupling is beyond the scope of the present work and has already been thoroughly investigated in the literature, the value reported in Ref. [10] is adopted in the quantum dynamics.

To estimate the magnitude of short-range effects in the couplings between P_{D1}-Chl_{D1} and P_{D2}-Chl_{D1}, the Coulombic contribution to the coupling - commonly considered the dominant term - was computed using the TrESP method [12]. Gas-phase excited-state calculations were performed with Gaussian 16 [13], while transition charges were obtained using Multiwfn [14]. The Coulombic coupling was then calculated as follows:

$$V_{\mathrm{TrESP}} = \frac{1}{4\pi\epsilon_0} \sum_i \sum_j \frac{q_i q_j}{R_{ij}} \tag{9}$$

Here q_i and q_j denote the transition charge for i^{th} and j^{th} atoms in two chromophores, respectively, with the interatomic distance R_{ij}. For all calculations, the ωB97XD [15, 16] and Def2-SVP [17, 18] have been used.

In Table Suppl. Table 2 the results obtained with both methods and for both dimers are reported. The calculations were performed on 11 different structures of the P_{D1}-P_{D2}-Chl_{D1} trimer

extracted every 20 ns from the ground-state trajectory and subsequently optimized at the QM-MM level, as described in Ref. [19]. The results show that the calculated couplings exhibit limited fluctuations along the trajectory. This observation is consistent with the small variations in the relative distances and orientations among the three pigments. The average center-of-mass distances over the 11 structural dimers of P_{D1}-P_{D2}, P_{D1}-Chl_{D1} and P_{D2}-Chl_{D1} are respectively 8.1, 10.6 and 12.3 Å; while the sigma are 0.25, 0.12 and 0.15 Å respectively. The angles between the chlorophylls rings planes are respectively 178.9°, 63.2° and 118.1°, with sigma of 1.1°, 1.5° and 1.8°. In addition, the results indicate that the inclusion of short-range contributions has only a minor effect. In particular, the short-range component accounts for approximately 8% of the P_{D1}-Chl_{D1} coupling and 3% of the P_{D2}-Chl_{D1} coupling. The couplings calculated here are consistent with those previously obtained using the TrESP approach [20, 2]. The previous values are slightly lower; however, they were also scaled by an empirical factor to account for solvent screening effects.

Timestep(ns)	$\lvert J_1^{\mathrm{TrESP}}\rvert$ (cm^{-1})	$\lvert J_1^{\mathrm{FED}}\rvert$ (cm^{-1})	$\lvert J_2^{\mathrm{TrESP}}\rvert$ (cm^{-1})	$\lvert J_2^{\mathrm{FED}}\rvert$ (cm^{-1})
0	61.4	66.2	79.0	81.4
20	58.8	64.5	64.5	65.3
40	59.0	62.9	68.9	70.3
60	57.9	62.5	74.6	77.4
80	57.7	62.0	86.3	90.4
100	64.0	69.3	77.0	80.2
120	61.4	67.2	73.6	76.2
140	67.3	71.6	69.3	71.3
160	64.4	69.5	80.4	83.9
180	59.8	64.1	69.2	70.2
200	68.1	73.8	68.1	68.2
Average	61.8 ± 1.1	66.7 ± 1.2	74.0 ± 2.0	75.9 ± 2.3

Suppl. Table 2: Absolute values of P_{D1}-Chl_{D1} and P_{D2}-Chl_{D1} couplings, named respectively J_1 and J_2, calculated with the TrESP and FED methods. The reported couplings have been calculated on different structures extracted every 20 ns from the ground 200 ns MM-MD of Photosystem-II.

Suppl. Note 2 Transition Energy (TE) calculation

Here we provide a brief description - along with system-specific technical details - of the procedure used to investigate the primary charge separation within the reaction center within the Perturbed Matrix Method (PMM) framework. For a more comprehensive theoretical discussion, the reader is referred to the relevant literature [21, 22].

In this study, we focus on the primary charge separation occurring in the $\mathrm{Chl_{D1}}$ channel, with the chlorophyll as donor and pheophytine as acceptor:

$$\mathrm{Chl_{D1}}^* + \mathrm{Pheo_{D1}} \rightarrow \mathrm{Chl_{D1}}^+ + \mathrm{Pheo_{D1}}^- \tag{10}$$

In line with our previous work [19], we first compute the unperturbed (i.e., gas-phase) quantum-mechanical properties of the chromophores involved. The QM calculations are carried out on individual chromophores (chlorophyll or pheophytin), treated as separate quantum centers rather than as a unique quantum system. The initial geometries are obtained through QM/MM optimization with electrostatic embedding, as implemented in ORCA [23]. Subsequently, the energies, dipole moments, and vertical excitation energies of the isolated chromophores are calculated using time-dependent density functional theory (TD-DFT) [24], employing the ωB97X-V functional [15, 25] and the Def2-TZVP basis set [17, 18], within the same computational framework as in Ref. [19]. These properties are computed for the donor chromophore in both its excited and oxidized states, and for the acceptor in its neutral and reduced forms. Then, the environment perturbation is applied to both the excited and oxidized states of the donor ($\mathrm{Chl_{D1}}$) as well as to the neutral and reduced states of the acceptor ($\mathrm{Pheo_{D1}}$) making use of the MD-PMM perturbation operator $\hat{V}_k$ for environment's configuration k (see also Eq. 4 in the main text):

$$\hat{V}_k \approx q_T \mathcal{V}_k(\mathbf{r}_0) - \mathbf{E}_k(\mathbf{r}_0) \cdot \hat{\boldsymbol{\mu}} \tag{11}$$

where q_T, $\mathbf{r_0}$, and $\hat{\boldsymbol{\mu}}$ are the QC's total charge, center of mass, and dipole operator, respectively; $\mathcal{V}_k$ is the electrostatic potential exerted by the perturbing environment's configuration k and $\mathbf{E}_k$ is the corresponding perturbing electric field ($\mathbf{E} = -\partial\mathcal{V}/\partial\mathbf{r}$).

Diagonalization of the perturbed Hamiltonian including the perturbation operator (see Eq. 5 in the main text) provides the perturbed energies for the donor in its excited and oxidized states ($\epsilon_{\mathrm{Chl}^*_{\mathrm{D1}}}$ and $\epsilon_{\mathrm{Chl}^+_{\mathrm{D1}}}$) and for the acceptor in its neutral and reduced states ($\epsilon_{\mathrm{Pheo_{D1}}}$ and $\epsilon_{\mathrm{Pheo}^-_{\mathrm{D1}}}$). Then, the transition energy (TE) for each k-th configuration is calculated as the energy difference between the perturbed energies of the donor/acceptor pair in the product state and in the reactant state.

$$\mathrm{TE}_k = [\epsilon_{\mathrm{Pheo}^-_{\mathrm{D1}}} + \epsilon_{\mathrm{Chl}^+_{\mathrm{D1}}}]_k - [\epsilon_{\mathrm{Pheo_{D1}}} + \epsilon_{\mathrm{Chl}^*_{\mathrm{D1}}}]_k \tag{12}$$

It is important to note that, within this semi-classical simulation framework, no actual charge transfer occurs. Rather, the simulation identifies the potential condition necessary for charge separation (the sudden degeneracy between the two aforementioned states) which corresponds to a TE value of zero. This estimate is finally corrected by applying an adiabaticity factor, previously computed in Ref [19] using the Landau-Zener approximation [22].

To investigate the origin of both the instantaneous and the $\langle\mathrm{TE}\rangle$ fluctuations, we focus on the electrostatic potential ($\mathcal{V}$) exerted on the exchanging porphyrins by the environment (see main text). As shown in Table Suppl. Table 3, $\mathcal{V}$ is by far the dominant term in the perturbation operator. In addition, as shown in Fig. Suppl. Figure 1, it is strongly correlated with the TE. In Table Suppl. Table 3 the electrostatic potential contributions from each portion of the system are also reported,

Suppl. Table 3: Average value of the perturbation term ($\hat{V}_k$) to the transition energy and its main components: total system potential ($q\mathcal{V}$), protein ($q\mathcal{V}_{\text{prot}}$), solvent+ions ($q\mathcal{V}_{\text{solvent+ions}}$) and the membrane bilayer ($q\mathcal{V}_{\text{membrane}}$).

$\langle q\mathcal{V} - \mathbf{E}\cdot\boldsymbol{\mu}\rangle$	$\langle q\mathcal{V}\rangle$	$\langle q\mathcal{V}_{\text{prot}}\rangle$	$\langle q\mathcal{V}_{\text{solvent+ions}}\rangle$	$\langle q\mathcal{V}_{\text{membrane}}\rangle$
-187 ± 15 kj/mol	-177 ± 16 kj/mol	-386 ± 13 kj/mol	192 ± 10 kj/mol	17 ± 10 kj/mol

showing that the electrostatic potential exerted by the protein is the major component in terms of magnitude. Such a contribution is partially compensated by the contribution arising from ions and solvent with a minor additional contribution from the membrane bilayer.

Suppl. Note 3 UV-Vis spectra calculation

The protocol to calculate the UV-Vis spectra using the PMM framework has been already applied and discussed in detail previously [26, 27]. Here we underline the technical details applied to this specific case. The quantum properties of the entire excitable trimer composed by $P_{D1}P_{D2}Chl_{D1}$ were calculated using the same approach described in the previous section. In particular, we included in the TD-DFT calculation the first three excited states. Thereafter, we perturbed these properties using the configurations of the 200 ns ground-state classical MD trajectory. Thus, we calculated the distribution of each perturbed excited state with an appropriate number of bins in the frequency space to grant a smooth but detailed curve. From these distributions we calculated the molar extinction coefficient $\varepsilon_{0,i}$ for the ground to the ith excited state transition, providing the effective absorption spectra (within the vertical transition approximation):

$$\varepsilon_{0,i}(\nu) \cong \sum_{\nu_{\text{ref}}} \frac{\Gamma_A(\nu_{\text{ref}})n(\nu_{\text{ref}})\text{h}\nu}{N} \frac{e^{-\frac{(\nu-\nu_{\text{ref}})^2}{2\sigma^2}}}{\sigma\sqrt{2\pi}} \tag{13}$$

$$\Gamma_A(\nu_{\text{ref}}) = \frac{|\boldsymbol{\mu}_{0,i}|^2_{\nu_{\text{ref}}}}{6\epsilon_0 c\hbar^2} \tag{14}$$

In these equations, ν_{ref}, $\text{n}(\nu_{\text{ref}})$ and $|\boldsymbol{\mu}_{0,i}|^2_{\nu_{\text{ref}}}$ are the frequency at the center of each bin, the corresponding number of MD frames and the transition dipole square norm, respectively. Additionally, $\hbar = \text{h}/(2\pi)$ with h the Planck constant, ϵ_0 is the vacuum dielectric constant, c is the light speed and σ^2 is the variance accounting for the semi-classical vibrations around the given reference structure. A multiplicative energy factor of 1.025 was used to align the maximum of the calculated band with the experimental peak which is centered at around 672 nm.

Suppl. Note 3.1 Excited state analysis

In order to characterize the nature of the low-lying excited states of the trimer, a fragment-based analysis of the one-electron transition density matrix (1TDM) was performed using the TheoDORE package [28, 29]. We define the fragments SP and Chl_{D1}.

For molecular systems composed of two (or more) chemically distinct regions or fragments, the electronic character of excited states can be analyzed using a fragment-based representation of the 1TDM for a transition from the ground state to the nth excited state. Within this framework, the transition from the ground state $|0\rangle$ to the $|n\rangle$ excited state is described by the transition density

matrix elements expressed in a basis of localized orbitals. The corresponding 1TDM element can be written as [29, 30, 31, 32]:

$$\hat{D}_{rs}^{0n} = \langle 0|\widehat{E}_{rs}|n\rangle \tag{15}$$

where $\widehat{E}_{rs}$ is the excitation operator involving the r and s orbitals.

The charge transfer number matrix element Ω_{AB}^{n} is defined by summing up the contributions from the regions A and B of the system [29].

$$\Omega_{AB}^{n} = \frac{1}{2}\sum_{\mu\in A}\sum_{\nu\in B}\left(\mathbf{D}^{0n}\mathbf{S}\right)_{\mu\nu}\left(\mathbf{S}\mathbf{D}^{0n}\right)_{\mu\nu} + D_{\mu\nu}^{0n}\left(\mathbf{S}\mathbf{D}^{0n}\mathbf{S}\right)_{\mu\nu} \tag{16}$$

where $\mathbf{S}$ is the atomic orbital overlap matrix and the summations run over basis functions associated with atoms belonging to fragments A and B.

For $A \neq B$, the quantity Ω_{AB}^{n} represents the weight of charge transfer (CT) from fragment A to fragment B, whereas diagonal elements ($A = B$) correspond to locally excited (LE) contributions on fragment A. The overall charge transfer character for the nth excited state is defined as the sum of all off-diagonal elements of the charge transfer number matrix [29]:

$$\mathrm{CT}^{n} = \frac{1}{\Omega^{n}}\sum_{A\neq B}\Omega_{AB}^{n}, \tag{17}$$

where $\Omega^{n} = \sum_{A,B}\Omega_{AB}^{n}$ is a normalization factor.

The CT descriptor thus reflects the cumulative weight of configurations in which the hole and the electron are localized on different fragments. Values of CT^{n} close to zero indicate predominantly local (Frenkel-type) excitations, whereas values approaching unity correspond to completely charge separated states.

In addition to the CT character, the participation ratio PR was evaluated in order to quantify the degree of exciton delocalization across the molecular fragments. The PR is defined from the charge transfer number matrix as [29]

$$\mathrm{PR}^{n} = \frac{1}{2}\left(\mathrm{PR}_{H}^{n} + \mathrm{PR}_{E}^{n}\right) \tag{18}$$

where the hole (H) and electron (E) participation ratios are defined as

$$\mathrm{PR}_{H}^{n} = \frac{\left(\Omega^{n}\right)^{2}}{\sum_{A}\left(\sum_{B}\Omega_{AB}^{n}\right)^{2}} \tag{19}$$

$$\mathrm{PR}_{E}^{n} = \frac{\left(\Omega^{n}\right)^{2}}{\sum_{B}\left(\sum_{A}\Omega_{AB}^{n}\right)^{2}} \tag{20}$$

and provides an estimate of the effective number of fragments involved in the excitation. PR values close to unity indicate localization on a single fragment, while values approaching two correspond to delocalization over two fragments.

Finally, the localization of the hole and electron was analyzed by evaluating the fragment-resolved hole (H) and electron (E) populations, obtained by integrating the corresponding densities over the SP and $\mathrm{Chl}_{\mathrm{D1}}$ fragments. These quantities provide complementary insight into the spatial distribution of the charge carriers following excitation and allow for a detailed interpretation of the excited-state character.

Excited state	ΔE (eV)	CT character	PR
S1	1.873	0.002	1.663
S2	1.884	0.001	1.044
S3	1.911	0.002	1.611
S4	2.385	0.003	1.049
S5	2.393	0.001	1.021

Suppl. Table 4: Vertical excitation energies (ΔE), charge-transfer (CT) character, and participation ratio (PR) for the five lowest singlet excited states.

Excited state	H (SP)	E (SP)	H (Chl_{D1})	E (Chl_{D1})
S1	0.286	0.287	0.757	0.756
S2	1.019	1.019	0.022	0.022
S3	0.777	0.777	0.265	0.265
S4	0.024	0.025	1.005	1.004
S5	1.017	1.016	0.011	0.011

Suppl. Table 5: Fragment-resolved hole (H) and electron (E) populations for the five lowest singlet excited states.

Suppl. Note 4 Kinetic Model of the Reaction Scheme

To model the kinetic scheme of the system, we considered seven microscopic events (Fig. 7A in the main text): the fast and slow charge separation (CS) processes, both originating from $\mathrm{Chl_{D1}}^*$ and leading to the formation of the $\mathrm{Chl_{D1}}^+\mathrm{Pheo_{D1}}^-$ radical pair (referred to as RP hereafter), with corresponding rate constants k_1 (fast) and k_2 (slow). We also included electronic energy transfer (EET) between SP* and $\mathrm{Chl_{D1}}^*$ within both the fast and slow excited-state compartments. These processes are characterized by rate constants k_3=k_4 for the EET from $\mathrm{Chl_{D1}}^*$ to SP*, and k_{-3}=k_{-4} for the corresponding back-reaction. Finally, interconversion between the two conformational states was initially considered, with a rate constant k_5 on the order of tens of picoseconds. All rate constants were obtained as detailed in the main text.

$$\frac{d[\mathrm{Chl_{D1}}^*]_F}{dt} = -(k_1 + k_3 + k_5) \cdot [\mathrm{Chl_{D1}}^*]_F + k_{-3} \cdot [\mathrm{SP}^*]_F + k_5 \cdot [\mathrm{Chl_{D1}}^*]_S \tag{21}$$

$$\frac{d[\mathrm{Chl_{D1}}^*]_S}{dt} = -(k_2 + k_4 + k_5) \cdot [\mathrm{Chl_{D1}}^*]_S + k_{-4} \cdot [\mathrm{SP}^*]_S + k_5 \cdot [\mathrm{Chl_{D1}}^*]_F \tag{22}$$

$$\frac{d[\mathrm{RP}]}{dt} = k_1 \cdot [\mathrm{Chl_{D1}}^*]_F + k_2 \cdot [\mathrm{Chl_{D1}}^*]_S \tag{23}$$

$$\frac{d[\mathrm{SP}^*]F}{dt} = -(k_{-3} + k_5) \cdot [\mathrm{SP}^*]_F + k_3 \cdot [\mathrm{Chl_{D1}}^*]_F + k_5 \cdot [\mathrm{SP}^*]_S \tag{24}$$

$$\frac{d[\mathrm{SP}^*]_S}{dt} = -(k_{-4} + k_5) \cdot [\mathrm{SP}^*]_S + k_4 \cdot [\mathrm{Chl_{D1}}^*]_S + k_5 \cdot [\mathrm{SP}^*]_F \tag{25}$$

Here, $[\mathrm{Chl_{D1}}^*]_F$ and $[\mathrm{Chl_{D1}}^*]_S$ represent the populations of the excited $\mathrm{Chl_{D1}}^*$ state in the fast and slow ET regimes, respectively. [RP] corresponds to the population of the CS intermediate $\mathrm{Chl_{D1}}^+$ $\mathrm{Pheo_{D1}}^-$ radical pair. $[\mathrm{SP}^*]_F$ and $[\mathrm{SP}^*]_S$ represent the populations of the excited SP* in the fast and slow regimes, respectively.

The time evolution of the population vector $\mathbf{P}(t)$ obeys

$$\frac{d\mathbf{P}}{dt} = \mathbf{M}\,\mathbf{P}, \tag{26}$$

where the 5×5 rate matrix is

$$\mathbf{M} = \begin{pmatrix} -(k_{-3}+k_5) & k_3 & k_5 & 0 & 0 \\ k_{-3} & -(k_3+k_1+k_5) & 0 & k_5 & 0 \\ k_5 & 0 & -(k_{-4}+k_5) & k_4 & 0 \\ 0 & k_5 & k_{-4} & -(k_4+k_2+k_5) & 0 \\ 0 & k_1 & 0 & k_2 & 0 \end{pmatrix}, \tag{27}$$

with rate constants $k_{-3} = k_{-4} = (296\ \mathrm{fs})^{-1}$, $k_3 = k_4 = (928\ \mathrm{fs})^{-1}$, $k_1 = (163\ \mathrm{fs})^{-1}$, $k_2 = (2000\ \mathrm{fs})^{-1}$, and $k_5 = (20 - 40\ \mathrm{ps})^{-1}$. The zero eigenvalue of $\mathbf{M}$ corresponds to probability conservation. The remaining four eigenvalues yield the apparent time constants τ_A, τ_B,τ_C,τ_D. Initial populations, as specified in the main text, were set to:

- $[\mathrm{Chl}^*_{\mathrm{D1}}]_F = 29\%$
- $[\mathrm{Chl}^*_{\mathrm{D1}}]_S = 20\%$
- $[\mathrm{RP}] = 0\%$

- $[SP^*]_F = 31\%$
- $[SP^*]_S = 20\%$

The excited-state calculations on the basis of which this assignment was made are provided in section Suppl. Note 3.1.

Since k_5 is significantly slower than k_1, k_2, $k_{3,4}$ and $k_{-3,-4}$ the interconversion between conformational states, underlying the fast and slow CS processes, can therefore be neglected from the differential equations. The two basins can be thus treated as kinetically independent. Numerical diagonalization of the full 5×5 matrix (Table Suppl. Table 6) confirms that halving k_5 from (20 ps)$^{-1}$ to (40 ps)$^{-1}$ shifts τ_A, τ_B, and τ_C by less than 3 fs, while τ_D, the only lifetime comparable to k_5^{-1} itself, shifts by approximately 150 fs (6%), .

Suppl. Table 6: Apparent time constants (fs) from numerical diagonalization of the full 5×5 rate matrix, compared with the analytical 2×2 eigenvalues, for two values of the interconversion rate k_5.

Method	τ_A	τ_B	τ_C	τ_D
Analytical 2×2 (fast basin)	125	–	386	–
Analytical 2×2 (slow basin)	–	218	–	2716
Numerical 5×5, $k_5 = (20\,\text{ps})^{-1}$	124	216	379	2396
Numerical 5×5, $k_5 = (40\,\text{ps})^{-1}$	125	217	382	2545

For each basin b the two-state system $SP^* \underset{k_b}{\overset{k_{-b}}{\rightleftharpoons}} Chl^*_{D1} \xrightarrow{k_b^{CT}} RP$ is described by the rate matrix:

$$\mathbf{M}^{(b)} = \begin{pmatrix} -k_{-b} & k_b \\ k_{-b} & -(k_b + k_b^{\text{CT}}) \end{pmatrix} \tag{28}$$

with eigenvalues given by:

$$\lambda_{\pm}^{(b)} = \frac{-(k_{-b} + k_b + k_b^{\text{CT}}) \pm \sqrt{(k_{-b} + k_b + k_b^{\text{CT}})^2 - 4\,k_{-b}\,k_b^{\text{CT}}}}{2} \tag{29}$$

The eigenvector associated with $\lambda_-^{(b)}$ (smaller eigenvalue, slower time constant) $|\lambda_-| < |\lambda_+|$) has components $(k_b,\ \lambda_-^{(b)} + k_{-b})$. Since $\lambda_-^{(b)} + k_{-b} > 0$ (both components positive), SP^* and $Chl_{D1}{}^*$ co-decay, identifying this as the CT1 mode. The eigenvector associated with $\lambda_+^{(b)}$ has components $(k_b,\ \lambda_+^{(b)} + k_{-b})$. Since $\lambda_+^{(b)} + k_{-b} < 0$ (opposite signs), SP^* and $Chl_{D1}{}^*$ evolve in anti-phase, identifying this as the EET equilibration mode. Therefore, the calculated time contants can be summarized as follows (the notation in parenthesis is used in the main text).
For the fast basin:

- τ_A=125 fs is associated with the EET process ($\tau_{\text{fast}}^{\text{EET}}$)
- τ_C=386 fs is associated with the CT process ($\tau_{\text{fast}}^{\text{CT1}}$)

For the slow basin:

- τ_B=218 fs is associated with the EET process ($\tau_{\text{slow}}^{\text{EET}}$)
- τ_D=2716 fs is associated with the CT process ($\tau_{\text{slow}}^{\text{CT1}}$)

Supplementary Figures

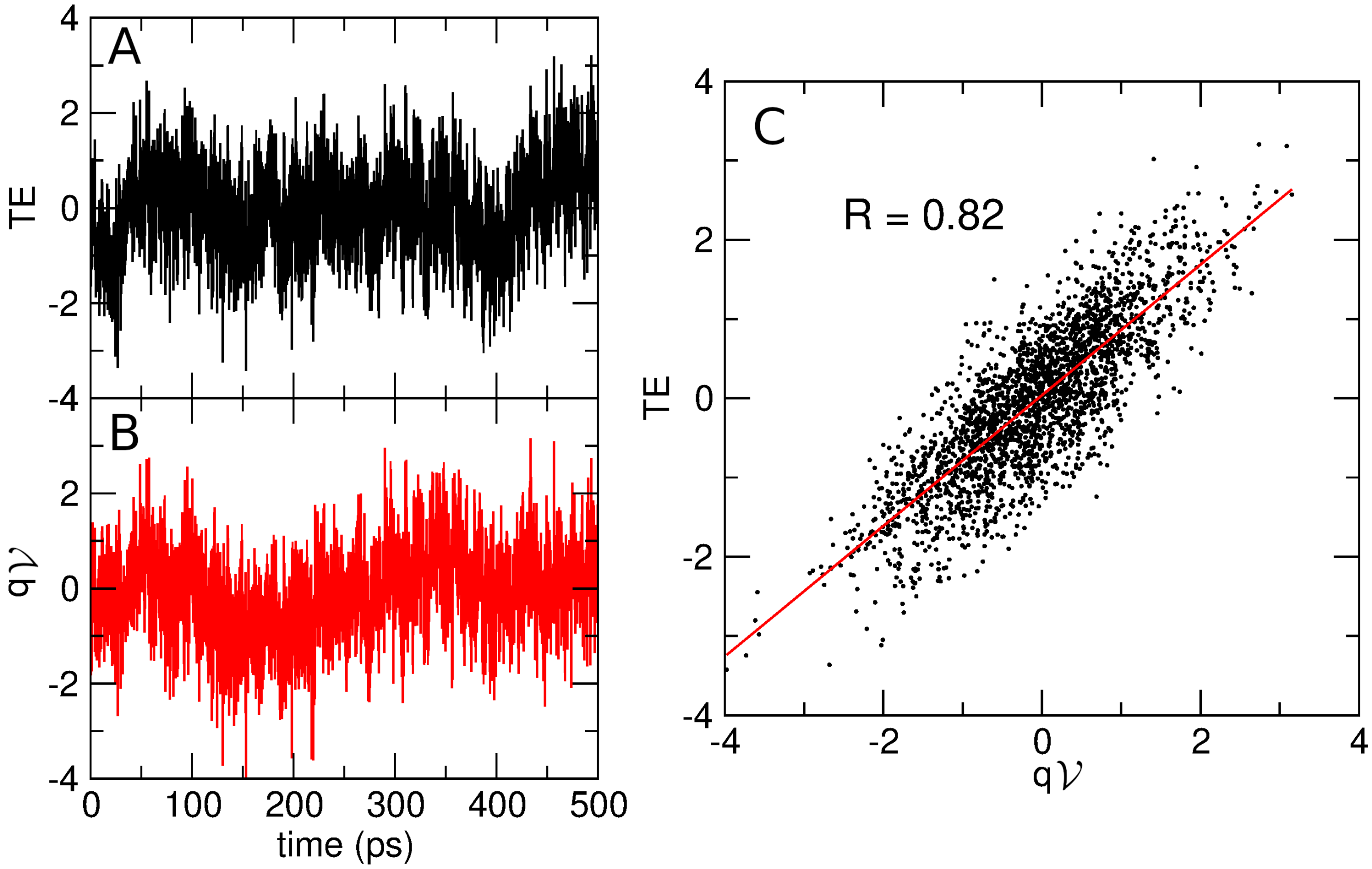

Suppl. Figure 1: Time evolution of the transition energy (TE, panel A) and electrostatic potential contribution to the transition energy ($q\mathcal{V}$, panel B) without the inclusion of the contribution of the counterions. In panel C, the correlation between the quantities is shown. Both TE and $q\mathcal{V}$ are normalized to be dimensionless, with zero mean and unit variance.

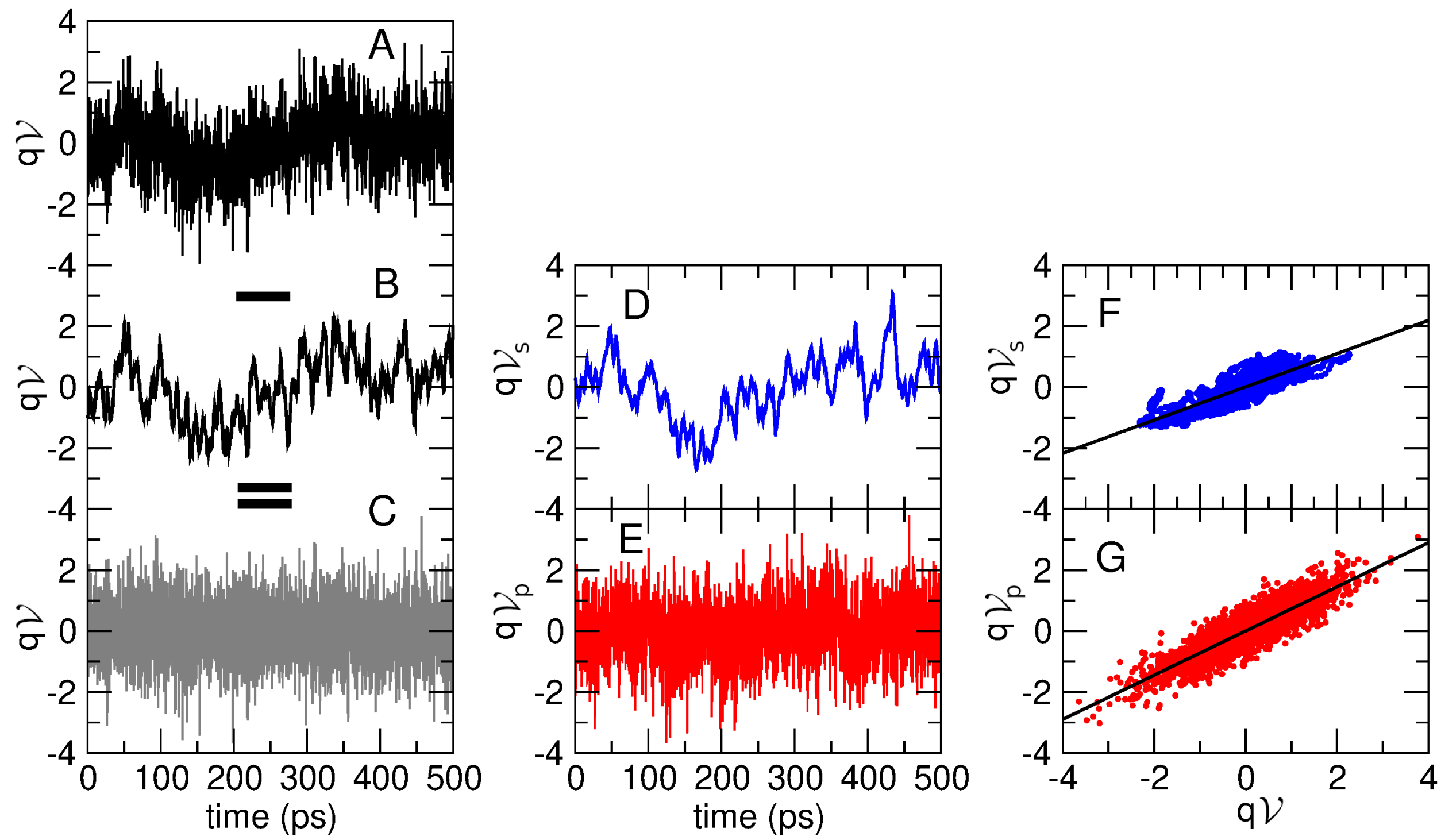

Suppl. Figure 2: Correlation between the instantaneous and $q\mathcal{V}$ fluctuations with the fluctuations of $q\mathcal{V}_p$ and $q\mathcal{V}_s$, respectively. A: time evolution of $q\mathcal{V}$; B: time evolution of $q\mathcal{V}$ in which fast fluctuations are filtered by performing a rolling average; C: time evolution of $q\mathcal{V}$ in which slow fluctuations are filtered (obtained by subtracting the trend in panel B from that in panel A). D: time evolution of $q\mathcal{V}_s$ in which fast fluctuations are filtered by performing a rolling average; E: time evolution of $q\mathcal{V}_p$. F: correlation between $q\mathcal{V}_s$ and $q\mathcal{V}$ both with fast fluctuations filtered ($R \approx 0.8$). G: correlation between $q\mathcal{V}_p$ and $q\mathcal{V}$ with slow fluctuations filtered ($R \approx 0.8$). All quantities are normalized to be dimensionless, with zero mean and unit variance.

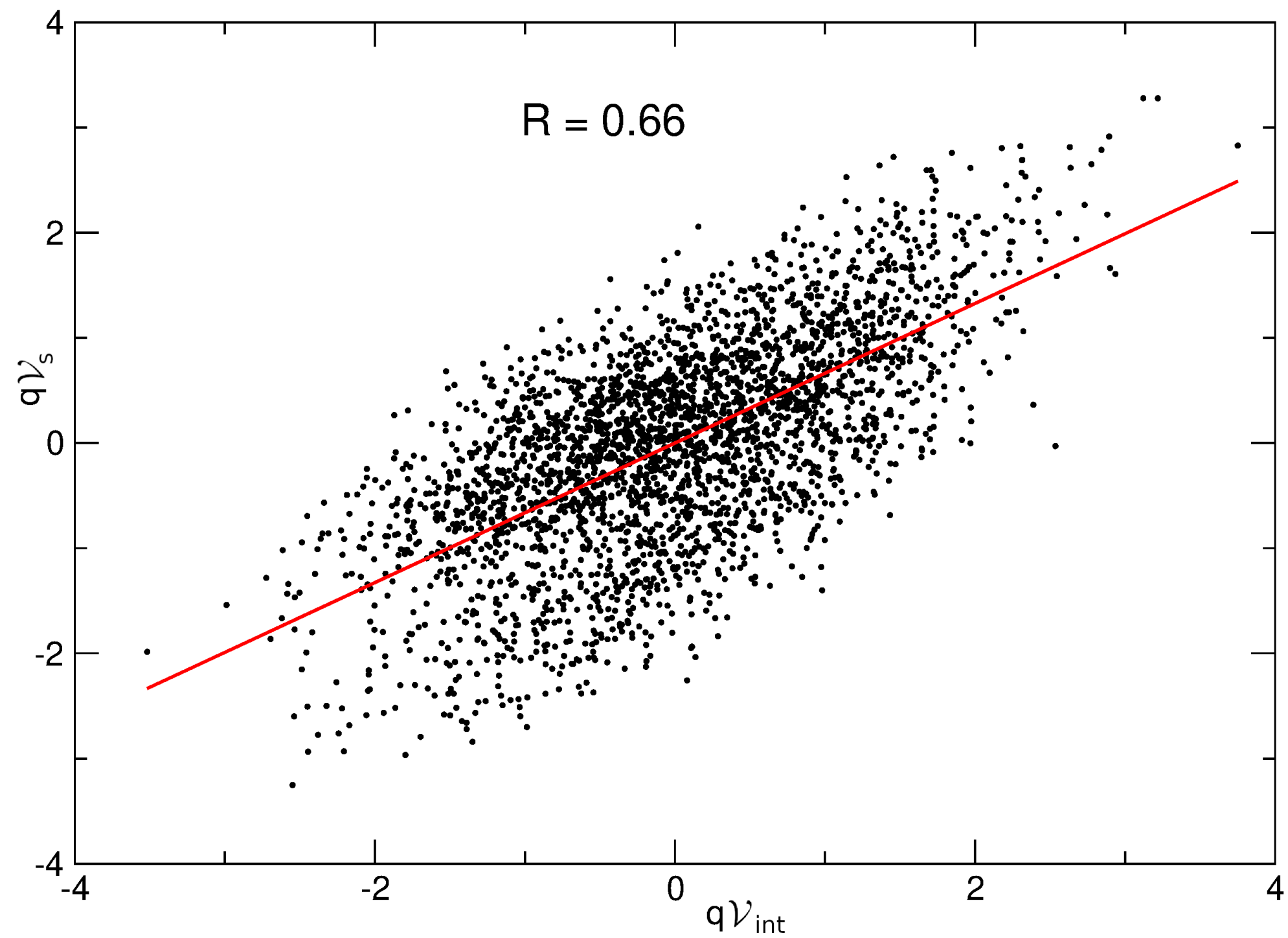

Suppl. Figure 3: Correlation between $q\mathcal{V}_s$ and the electrostatic potential exerted by the internal water molecules (i.e., water molecules within 25 Å of $\mathrm{Chl_{D1}}$ and $\mathrm{Pheo_{D1}}$ and belonging to the OEC channels) $q\mathcal{V}_{int}$. Both quantities are normalized to be dimensionless, with zero mean and unit variance.

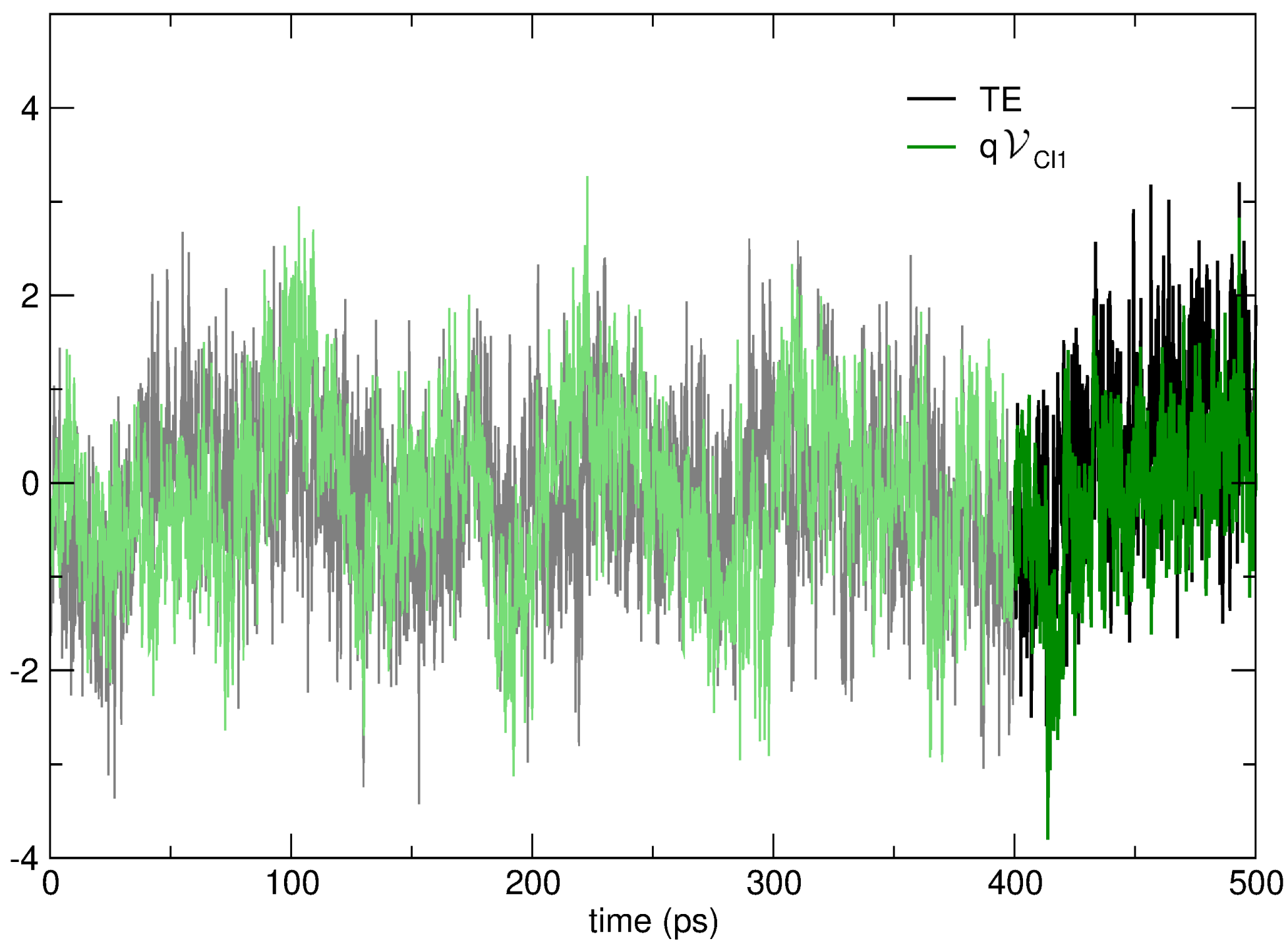

Suppl. Figure 4: Time evolution of TE and the electrostatic potential exerted by the water molecules belonging to the Cl1 channel ($q\mathcal{V}_{\mathrm{Cl1}}$). Both quantities are normalized to be dimensionless, with zero mean and unit variance.

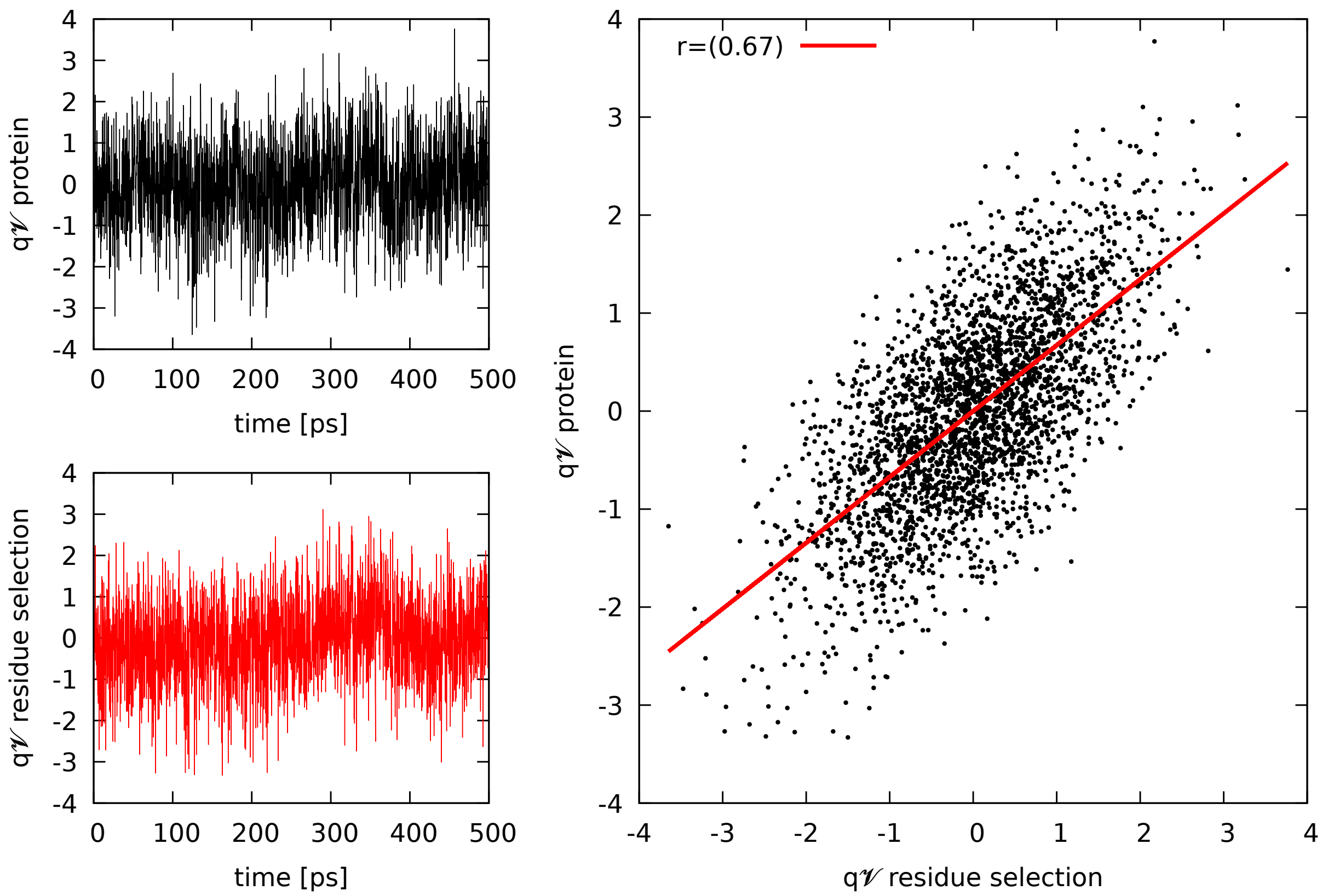

Suppl. Figure 5: Left panels: time evolution of the protein electrostatic potential (top) and sum of the protein electrostatic potential of selected residues from the first three eigenvectors calculated with ED analysis (bottom). Right panel: correlation between $q\mathcal{V}$ of the protein and $q\mathcal{V}$ of the residue selection. Both quantities are normalized to be dimensionless, with zero mean and unit variance.

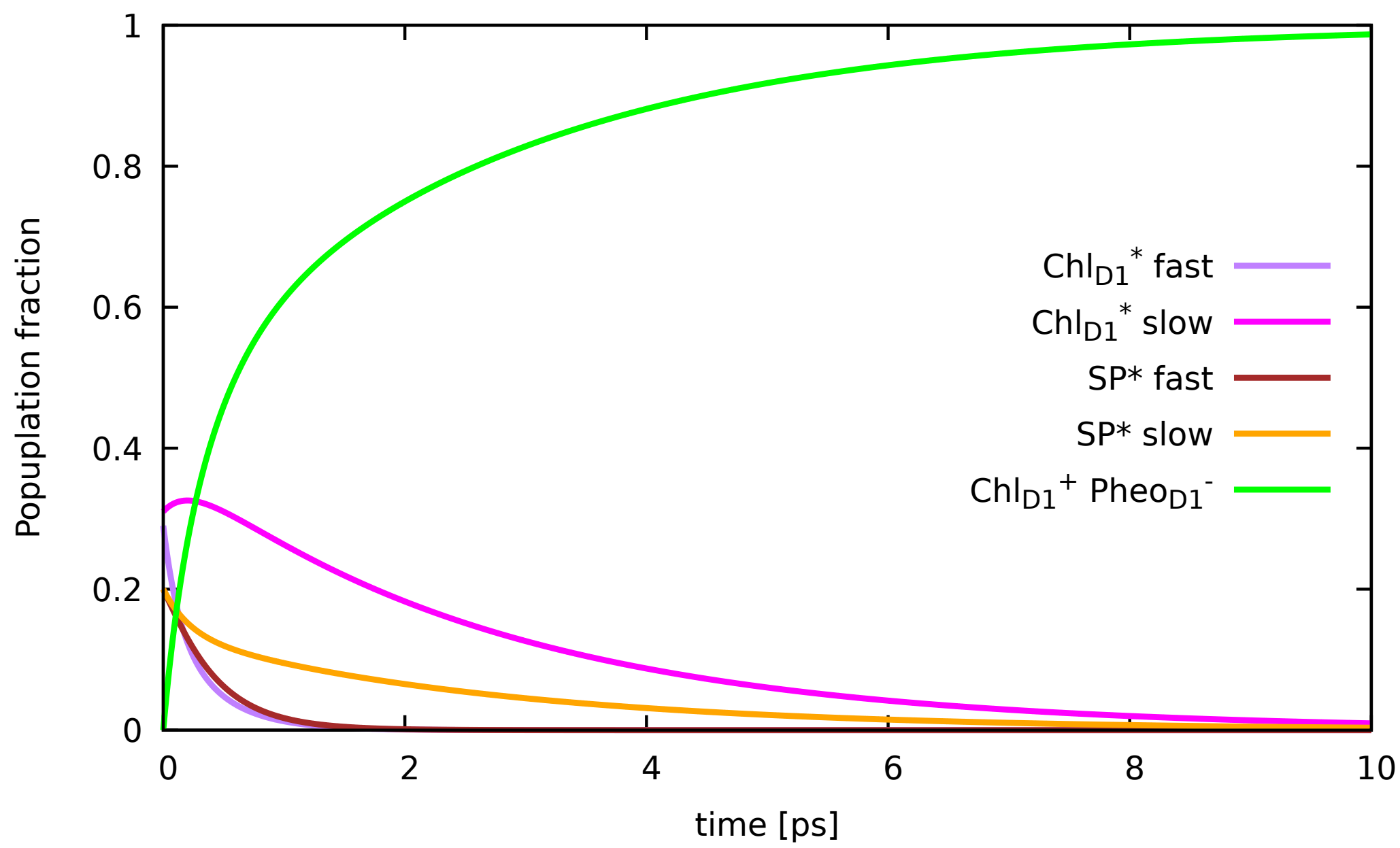

Suppl. Figure 6: Time evolution of the relative populations of the key species involved in excitation energy transfer and charge transfer processes obtained by solving the system of kinetic equations. The species are: Chl^*_{D1} in a fast (violet) and slow (magenta) regime; SP* in a fast (brown) and slow (orange) regime; and the radical pair $Chl^+_{D1}Pheo^-_{D1}$ (green).

Supplementary References

[1] Richard Stones et al. “On the performance of a photosystem II reaction centre-based photocell”. en. In: *Chem. Sci.* 8.10 (Oct. 2017), pp. 6871–6880.

[2] Grzegorz Raszewski et al. “Spectroscopic properties of reaction center pigments in photosystem II core complexes: revision of the multimer model”. en. In: *Biophys. J.* 95.1 (July 2008), pp. 105–119.

[3] Grzegorz Raszewski and Thomas Renger. “Light harvesting in photosystem II core complexes is limited by the transfer to the trap: can the core complex turn into a photoprotective mode?” In: *J. Am. Chem. Soc.* 130.13 (2008), pp. 4431–4446.

[4] Jiajun Ren et al. “Time-dependent density matrix renormalization group method for quantum dynamics in complex systems”. en. In: *Wiley Interdiscip. Rev. Comput. Mol. Sci.* 12.6 (Nov. 2022), e1614.

[5] Raffaele Borrelli and Maxim F Gelin. “Finite temperature quantum dynamics of complex systems: Integrating thermo-field theories and tensor-train methods”. en. In: *Wiley Interdiscip. Rev. Comput. Mol. Sci.* 11.6 (Nov. 2021), e1539.

[6] *Renormalizer*. `https://github.com/shuaigroup/Renormalizer`.

[7] A. A. Voityuk and N. Rösch. “Fragment charge difference method for estimating donor–acceptor electronic coupling: Application to DNA π-stacks”. In: *J. Chem. Phys.* 117 (2002), pp. 5607–5616.

[8] Chao-Ping Hsu, Zhi-Qiang You, and Hung-Cheng Chen. “Characterization of the short-range couplings in excitation energy transfer”. In: *The Journal of Physical Chemistry C* 112.4 (2008), pp. 1204–1212.

[9] Michele Nottoli et al. “The role of charge-transfer states in the spectral tuning of antenna complexes of purple bacteria”. In: *Photosynthesis research* 137.2 (2018), pp. 215–226.

[10] Felix G Gemeinhardt et al. “Short-range effects in the special pair of photosystem II reaction centers: The nonconservative nature of circular dichroism”. In: *The journal of physical chemistry letters* 14.51 (2023), pp. 11758–11767.

[11] Jing Kong et al. “Q-Chem 2.0: a high-performance ab initio electronic structure program package”. In: *Journal of Computational Chemistry* 21.16 (2000), pp. 1532–1548.

[12] Mohamed E Madjet, Ayjamal Abdurahman, and Thomas Renger. “Intermolecular Coulomb couplings from ab initio electrostatic potentials: application to optical transitions of strongly coupled pigments in photosynthetic antennae and reaction centers”. In: *The Journal of Physical Chemistry B* 110.34 (2006), pp. 17268–17281.

[13] M. J. Frisch et al. *Gaussian 16*. Gaussian Inc. Wallingford CT. 2016.

[14] Tian Lu and Feiwu Chen. “Multiwfn: A multifunctional wavefunction analyzer”. In: *J. Comp. Chem.* 33.5 (2012), pp. 580–592.

[15] Jeng-Da Chai and Martin Head-Gordon. “Long-range corrected hybrid density functionals with damped atom–atom dispersion corrections”. In: *Physical Chemistry Chemical Physics* 10.44 (2008), pp. 6615–6620.

[16] Jeng-Da Chai and Martin Head-Gordon. “Systematic optimization of long-range corrected hybrid density functionals”. In: *The Journal of chemical physics* 128.8 (2008).

[17] Florian Weigend and Reinhart Ahlrichs. "Balanced basis sets of split valence, triple zeta valence and quadruple zeta valence quality for H to Rn: Design and assessment of accuracy". In: *Phys. Chem. Chem. Phys.* 7.18 (2005), pp. 3297–3305.

[18] Florian Weigend. "Accurate Coulomb-fitting basis sets for H to Rn". In: *Phys. Chem. Chem. Phys.* 8.9 (2006), pp. 1057–1065.

[19] Matteo Capone et al. "Alternative Fast and Slow Primary Charge-Separation Pathways in Photosystem II". In: *Angew. Chem. Int. Ed.* 62.16 (2023), e202216276.

[20] Keisuke Saito et al. "Quantum mechanical analysis of excitation energy transfer couplings in photosystem II". In: *Biophysical Journal* 122.3 (2023), pp. 470–483.

[21] A Amadei, I Daidone, and M Aschi. "A general theoretical model for electron transfer reactions in complex systems". In: *Phys. Chem. Chem. Phys.* 14 (2012), pp. 1360–1370.

[22] Andrea Amadei and Massimiliano Aschi. "Theoretical-computational modeling of charge transfer and intersystem crossing reactions in complex chemical systems". In: *RSC Adv.* 8.49 (2018), pp. 27900–27918.

[23] Frank Neese et al. "The ORCA quantum chemistry program package". In: *J. Chem. Phys.* 152.22 (2020), p. 224108.

[24] Robert G Parr and Weitao Yang. "Density-functional theory of the electronic structure of molecules". In: *Annu. Rev. Phys. Chem.* 46.1 (1995), pp. 701–728.

[25] Narbe Mardirossian and Martin Head-Gordon. "ωB97X-V: A 10-parameter, range-separated hybrid, generalized gradient approximation density functional with nonlocal correlation, designed by a survival-of-the-fittest strategy". In: *Phys. Chem. Chem. Phys.* 16.21 (2014), pp. 9904–9924.

[26] Maira D'Alessandro et al. "Theoretical modeling of UV-Vis absorption and emission spectra in liquid state systems including vibrational and conformational effects: The vertical transition approximation". In: *J. Chem. Phys.* 139.11 (2013).

[27] Marco D'Abramo, Massimiliano Aschi, and Andrea Amadei. "Theoretical modeling of UV-Vis absorption and emission spectra in liquid state systems including vibrational and conformational effects: Explicit treatment of the vibronic transitions". In: *J. Chem. Phys.* 140.16 (2014).

[28] Felix Plasser. "TheoDORE: A toolbox for a detailed and automated analysis of electronic excited state computations". In: *The Journal of chemical physics* 152.8 (2020).

[29] Felix Plasser and Hans Lischka. "Analysis of excitonic and charge transfer interactions from quantum chemical calculations". In: *Journal of chemical theory and computation* 8.8 (2012), pp. 2777–2789.

[30] Sergei Tretiak and Shaul Mukamel. "Density matrix analysis and simulation of electronic excitations in conjugated and aggregated molecules". In: *Chemical reviews* 102.9 (2002), pp. 3171–3212.

[31] AV Luzanov and OV Prezhdo. "Irreducible charge density matrices for analysis of many-electron wave functions". In: *International journal of quantum chemistry* 102.5 (2005), pp. 582–601.

[32] AV Luzanov and OA Zhikol. "Electron invariants and excited state structural analysis for electronic transitions within CIS, RPA, and TDDFT models". In: *International Journal of Quantum Chemistry* 110.4 (2010), pp. 902–924.
