# Supplementary material for: Water-modulated conformational heterogeneity underlies multiple timescales of primary charge separation in photosystem II
Source: Nat Commun. 2026 Jun 20;17:7795. doi: 10.1038/s41467-026-73961-w (PMC13439440; doi:10.1038/s41467-026-73961-w)
Supplement: Supplementary file 2 [file 41467_2026_73961_MOESM2_ESM.pdf]

The Description of Additional Supplementary Files

Supplementary data 1: input files, force field parameters, and starting and final structures of the classical MD simulations.

Supplementary data 2: QM/MM optimized structures of the trimer.
