# Supplementary material for: Water-modulated conformational heterogeneity underlies multiple timescales of primary charge separation in photosystem II
Source: Nat Commun. 2026 Jun 20;17:7795. doi: 10.1038/s41467-026-73961-w (PMC13439440; doi:10.1038/s41467-026-73961-w)
Supplement: Supplementary file 3 [file 41467_2026_73961_MOESM5_ESM.pdf]

nature portfolio

Peer Review File

Water-Modulated Conformational Heterogeneity Underlies Multiple Timescales of Primary Charge Separation in Photosystem II

Corresponding Author: Dr Dimitrios Pantazis

This file contains all reviewer reports in order by version, followed by all author rebuttals in order by version.

Version 0:

Reviewer comments:

Reviewer #1

(Remarks to the Author)
In this paper the authors propose a new interpretation of the dynamics in the PSII reaction centre. While this is of course interesting the paper is rather technical and not all data match experiment very well bringing doubt on the interpretation. Furthermore, insufficient information is given for others to reproduce the calculations. Below more detailed feedback is given, however, I would propose that the authors work out the findings in more detail, write up the manuscript in a way where the comparisons between experiment and theory is more transparent (for example reporting numbers in tables) and submit the paper to a more technical journal.

1. The authors assume the incoherent TRET equation for the transfer (Eq. 1). How clear it is that at least the fastest dynamics has no coherent components?

2. A table providing a concise overview of the average contributions to the transition
energy from the protein and the water would be useful.

3. How important are the decimals after the comma used for the initial populations for the conclusions here? Assuming a 40.2% population, for example, seems very accurate. Are the conclusions depending on this?

4. A large amount of numbers are distributed in the text instead of compared in figures and tables. This makes it very hard for the reader to compare, follow the discussion, and validate the conclusions.

5. The conclusion that the bi-exponential behaviour is due to water-modulated conformational heterogeneity is interesting but in conflict with conclusions of the same authors in the past and given the weak connection with experiment presented not clearly justified.

6. The Berendsen barostat is known to not reproduce the proper ensamble. Why was the Rahman-Parrinello barostat not use and what are the potential consequences? What was the simulation temperature and pressure?

7. The predicted spectra in Figure 7B match experiment poorly. How does the lack of predictive power of the used model affect the other properties and the conclusions?

8. Provide references for exchange correlation functional and basis set in section 1.1.

9. Provide the calculated TrESP charges in the SI.

10. Report the used transition dipoles in de PDA.

11. Repeat the values of the couplings given in the main in the table of SI1.1 for simplicity for the reader.

12. Why were different DFT functionals used for the TrESP and energy calculations?

13. Provide an explicit equation for the transition energy.

Reviewer #2

(Remarks to the Author)
The authors investigate charge separation (CS) in the Photosystem II (PSII) reaction center, focusing on the ChlD1$^+$ pathway through the ChlD1$^+$PheoD1$^-$ intermediate. They propose that structural changes in the protein and the surrounding water particularly dynamic water channels near the oxygen-evolving complex (OEC) help explain the different timescales observed in the two steps CS pathway: RC --> ChlD1$^+$PheoD1$^-$ --> SP$^+$PheoD1$^-$
To address ongoing debates about CS timescales, they use a multiscale computational approach combining molecular dynamics, QM/MM and the Perturbed Matrix Method (PMM). This allows them to model how environmental flexibility influences CS and they also apply Förster theory to estimate excitation energy transfer within the reaction center. Their model provides insight into how CS is modulated by water channel and protein conformational changes, supporting a refined interpretation of parallel pathways.
While the work is scientifically sound and likely publishable, I have some major concerns that the authors should address before acceptance:
1. Why did the authors choose to use TrESP exciton coupling to calculate couplings between the pigment pairs within the reaction center, which involves strong charge separation character? I disagree with the statement that "short-range coupling originating from the orbital overlap of frontier molecular orbitals might not have major contributions to the overall excitonic coupling. Hence, the short-range effects are neglected." When modeling coupling between charge separation states, a diabatization scheme should be considered. As validation, the authors could perform TD-DFT calculations in gas-phase on the special pair to compute the full coupling $V = 1/2\ (E1 - E2)$, where E1 and E2 are the first two excited state energies and then compare this to TrESP coupling values. I expect significant short-range orbital overlap effects in the coupling that would impact the kinetics.
2. Why was the Förster rate equation used to model population kinetics involving electron transfer? Marcus theory should be applied instead, as it better accounts for strong charge separation coupling within a diabatization framework.
3. Although the authors have demonstrated the effect of the protein on the charge separation state and its kinetics using a single structure (J. Am. Chem. Soc. 142, 18174–18190 (2020)) and dynamics with the PMM method (Angew. Chem. Int. Ed. 62, e202216276 (2023)), I wonder what new insights this study provides beyond the comprehensive analysis of the different charge separation pathways within the PSII-RC.
4. I am curious about the mechanism that enables such a long-distance charge separation state between SP$^+$ and PheoD1$^-$. Is this driven by strong HOMO-LUMO overlap or are other factors involved?

Minor points:
I think the crystal structure (3WU2) does not show the full PSII because it is missing the main antenna complex, LHCII. It looks like it only includes the PSII core (CP43 and CP47) and the reaction center (D1 and D2) complexes. I suggest the authors use PSII-RC instead of PSII when referring to the reaction center.

Reviewer #3

(Remarks to the Author)
In this manuscript the authors present a well-executed and conceptually strong study that addresses a long-standing question in photosynthetic charge-separation kinetics. The authors combine ultrafast kinetic modeling with detailed molecular dynamics simulations using the MD–Perturbed Matrix Method to elucidate the role of protein–solvent dynamics, particularly water-channel reorganization, in governing the observed multi-exponential behavior of primary charge separation.

The work is of high quality in several respects:
1) Scientific significance: The study provides a unifying explanation for disparate experimental observations and resolves a persistent debate regarding the origin of multiple time constants in Photosystem II.
2) Methodological rigor: The combination of extended atomistic MD simulations, hybrid quantum/classical treatment, and numerical kinetic modeling is thorough and well justified. The MD-PMM framework is applied appropriately, and the details provided inspire confidence in the reproducibility and validity of the results.
3) Clarity of presentation: The manuscript is well written, with the Introduction and Discussion clearly framing the context and implications of the findings. Figures and tables are clear, relevant, and informative.
4) Quality of results: The agreement between the simulated apparent lifetimes and experimental measurements is impressive. The link between structural fluctuations of water channels and kinetic heterogeneity is convincingly demonstrated.

The conclusions are fully supported by the data, and the manuscript advances our mechanistic understanding of ultrafast charge separation in a key photosynthetic complex. It will be of interest to researchers in photosynthesis, biophysics, and theoretical/computational chemistry. I strongly support publication of this manuscript in its present form. The study is a valuable contribution to the field and meets the high standards of the journal.

Reviewer #4

(Remarks to the Author)
This paper provides an important insight. The authors present a comprehensive model of primary charge separation, based on their theoretical calculations in reference to recent experimental results.
The work is worthy of publication. However, the problem itself is complex, and the available experimental results are often limited and partial. The reviewer would like to request further discussion as outlined below:

To what extent, and with what accuracy, can the current theoretical methods employed here be considered reliable? What are their limits of applicability? Convincing references to past literature, if available, are needed. This is critical in order to avoid arbitrary adjustments of the calculated results, given that experimental results vary from case to case.

Related to the above, the OEC is well known for its challenging spin states. In the present problem, which also involves radical states, how accurate are the theoretical methods?

Although the following question is somewhat philosophical, the reviewer would still like to know the authors' view. Figure 1B shows three possible pathways, and the authors focused on the $ChlD1^{+}$ pathway. Fundamentally, the phenomenon involves the selection of quantum mechanical states. Therefore, choosing a pathway corresponds to choosing the weight or amplitude of the states. Is this weight beyond the scope of the current method?

Minor questions

In the last phrase of Protein effect: "..., rather than a unique quantum center," the meaning is unclear. Could the authors clarify?

In Figure 4, panel A, the right-side graph is aligned with the arrays beside the slow-ET, although it should correspond to the fast-ET. Could the authors check this?

Version 1:

Reviewer comments:

Reviewer #1

(Remarks to the Author)
The authors have revised the manuscript based on the feedback from all reviewers. While most points improved a few misconceptions sneeked in. In the answer to the question regarding coherent components to the EET the authors now (in their answer and the manuscript) claim that a coherent component would speed up the transfer. This is a misconception. Equation (1) in the manusript is known to predict too fast transfer as the coherent limit is approached (see for example 10.1063/5.0136652, but undoubedtly this is also discussed in much older literature by for example Silbey and Reineger.) As the coupling increase the cross-over leads to a slower J dependence on the coupling instead of the J^2 one. Förster transfer provides an upper limit NOT a lower limit. A coherent component would slow it down as the next fourth-order perturbation theory has the opposite sign of the second-order. It would be better to demonatrate that the present parameters are in the regime where Eq. (1) is applicable for example by comparing with other methods (as also proposed by another reviewer).

The authors now report the simulation temperature and pressure. The used constants for the thermostat+barostat (time-scale and compressability) are not reported. I strongly recommend that the authors ensure that the results will be reproducible.

The authors removed the experimental data provided for comparizon. I would recommend not hiding it, but state clearly why the agreement can be considered sufficient for the purpose of the paper. I would avoid removing data which could be seen as cherry picking.

After addressing these issues the paper can potentially be recommended.

Reviewer #2

(Remarks to the Author)
The study is interesting and technically well executed, and I appreciate that the revised version attempts to address the reviewers' earlier comments. However, some fundamental methodological limitations remain, which in my view reduce the overall novelty and suitability of the work for Nature Communications.

The coupling between ChlD1 and SP* is still evaluated using the TrESP approach, assuming negligible charge-transfer or overlap contributions. In the PSII reaction center, however, exciton and charge-transfer mixing are commonly known to occur, and this assumption may not be fully justified for confirming the bi-exponential excitation pathways. To verify that short-range orbital overlap and charge-transfer effects are indeed minor, I would still recommend performing TD-DFT calculations (e.g., exciton splitting or CT character analysis) on representative pigment dimers. This would provide a firmer validation of the coupling strengths used in the kinetic model.

In addition, the PMM-MD methodology remains based on classical molecular dynamics sampling, which cannot capture nonadiabatic transitions or electronic coherence that likely influence ultrafast charge-separation dynamics. While PMM is

valuable for describing environmental fluctuations, it lacks the explicit quantum treatment of electronic motion available in mixed quantum-classical (MQC) approaches. This limitation should be discussed more explicitly, as it constrains both the mechanistic interpretation and the generality of the conclusions.

Overall, the manuscript presents a careful and informative study, but the methodological framework remains semi-classical, and the results largely represent an incremental extension of previous work by the same group. Thus, although the topic is of clear scientific interest, in my view, the manuscript may not yet reach the level of conceptual advance expected for Nature Communications.

Reviewer #4

(Remarks to the Author)
All the points that I requested are replied, I agree to access this paper.

Version 2:

Reviewer comments:

Reviewer #1

(Remarks to the Author)
The authors addressed all my previous comments and I think that the manuscript considerably improved.

Reviewer #2

(Remarks to the Author)
The authors have made a significant effort to address the reviewer comments. I am particularly pleased with the inclusion of full coupling calculations, including short-range contributions as well as the use of TD-DMRG quantum dynamics to account for electronic coherence. I am therefore happy to recommend this work for publication.

Open Access This Peer Review File is licensed under a Creative Commons Attribution 4.0 International License, which permits use, sharing, adaptation, distribution and reproduction in any medium or format, as long as you give appropriate credit to the original author(s) and the source, provide a link to the Creative Commons license, and indicate if changes were made.
In cases where reviewers are anonymous, credit should be given to 'Anonymous Referee' and the source.
The images or other third party material in this Peer Review File are included in the article’s Creative Commons license, unless indicated otherwise in a credit line to the material. If material is not included in the article’s Creative Commons license and your intended use is not permitted by statutory regulation or exceeds the permitted use, you will need to obtain permission directly from the copyright holder.
To view a copy of this license, visit https://creativecommons.org/licenses/by/4.0/

Responses to the reviewers' comments

Reviewer #1:

In this paper the authors propose a new interpretation of the dynamics in the PSII reaction centre. While this is of course interesting the paper is rather technical and not all data match experiments very well bringing doubt on the interpretation. Furthermore, insufficient information is given for others to reproduce the calculations. Below more detailed feedback is given, however, I would propose that the authors work out the findings in more detail, write up the manuscript in a way where the comparisons between experiment and theory is more transparent (for example reporting numbers in tables) and submit the paper to a more technical journal.

We thank the reviewer for the constructive feedback and for highlighting important points that have helped improve the manuscript. We have revised the manuscript to address the issues raised. In particular, we have clarified the comparison between experimental and calculated results (presenting numerical values in tables where appropriate) and have added more detailed information about the computational methodology to improve reproducibility. Below, we provide a detailed point-by-point response to each of the reviewer's comments.

1. The authors assume the incoherent TRET equation for the transfer (Eq. 1). How clear it is that at least the fastest dynamics has no coherent components?

The present study primarily focuses on the interplay between energy transfer and charge separation, which is a highly complex problem. We agree with the referee that, for the ultrafast energy transfer step we predict (k_{-4}=121 fs), coherence effects could play a role. Nevertheless, Förster theory offers a reasonable first approximation for estimating energy transfer rates. For consistency with the rest of our kinetic model, according to which coherence is not included in the electron transfer steps, we have therefore restricted our treatment to the widely used incoherent energy transfer formalism. While we acknowledge that our system is not strictly within the Förster limit, incorporating coherence would likely accelerate energy transfer. However, this does not alter our main conclusions regarding the order of magnitude of the apparent lifetimes identified by the kinetic model, as demonstrated by testing the effect of varying the fastest EET rate constant, k_{-4}, from 80 fs to 160 fs. The results are now provided in Table S3 in the Supplementary Information.

We also added the following paragraphs to the main text (page 4):

"*For the ultrafast energy transfer step (121 fs), coherence effects may contribute. Nevertheless, Förster theory provides a reasonable first approximation and is applied here for consistency with the rest of our kinetic model, which accounts for the interplay between energy transfer and charge separation, a highly complex process. Although the system may not lie strictly within the Förster limit, coherence would likely accelerate energy transfer, which would not alter our main conclusions regarding the order of magnitude of the apparent lifetimes identified by the kinetic model (see below).*"

And (page 10):

"*To assess the effect of varying the fastest rate constant (EET k_{-4}), which may be accelerated by coherence effects, on the calculated apparent time constants, we solved the differential equations for different k_{-4} values. The resulting apparent time constants, reported in Table S3 of the SI, show that the order of magnitude of all three constants remains unchanged.*"

2. A table providing a concise overview of the average contributions to the transition energy from the protein and the water would be useful.

As suggested by the reviewer, we have added a table to the revised Supporting Information (Table S2), reporting the average contributions of the protein and water to the total electrostatic potential which is in turn the major contribution to the transition energy as shown in the new table.

3. How important are the decimals after the comma used for the initial populations for the conclusions here? Assuming a 40.2% population, for example, seems very accurate. Are the conclusions depending on this?

As suggested by the reviewer, the results are not affected by the decimal digits of the initial populations. To avoid unintentionally implying otherwise, the initial populations have been approximated as rounded numbers in the revised version of the manuscript. In addition, we would like to point out that the apparent time constants, and therefore the conclusions drawn, do not depend on the initial population values, which only affect the amplitudes of the different decays.

4. A large amount of numbers are distributed in the text instead of compared in figures and tables. This makes it very hard for the reader to compare, follow the discussion, and validate the conclusions.

Following the reviewer's suggestion, we added to the kinetic model section a table in which our calculated apparent time constants are compared to experimental data measured on different timescales (Table 1). We have now rephrased the corresponding paragraph accordingly (page 10):

"*Nevertheless, the computed results are generally consistent with experimental observations, as shown in Table 1, which compares the apparent time constants obtained in this work with relevant data, giving confidence to the theoretical predictions.*"

5. The conclusion that the bi-exponential behaviour is due to water-modulated conformational heterogeneity is interesting but in conflict with conclusions of the same authors in the past and given the weak connection with experiment presented not clearly justified.

We thank the reviewer for raising this point. We respectfully believe that the conclusions presented in this work, specifically regarding the origin of the bi-exponential behavior of primary charge separation, are not in conflict with our previous publication (Angew. Chem. Int. Ed. 62, e202216276, 2023). In that earlier work, we already proposed a bi-exponential kinetic behavior for the same reaction. In the present study, we report an unexpected molecular explanation for the observed kinetics, attributing it to water-modulated conformational heterogeneity. We agree with the reviewer that this specific mechanistic interpretation does not have a direct connection with experimental data. However, we would like to emphasize that the calculated apparent time constants are in good agreement with the experimentally observed values. This consistency supports the soundness of our proposed mechanism. In general, the alignment between calculated and experimental observables is a significant foundation for using simulations to suggest molecular-level interpretations that may not be directly accessible through experiments alone.

We have now rephrases part of the conclusions to highlight this point (page 10):

"*We propose that the ChlD1+ pathway, i.e. the direct reduction of PheoD1 by the excited ChlD1, is the dominant charge-separation route, and that the bi-exponential kinetics instead reflect conformational heterogeneity within this pathway. This conclusion is supported by a detailed examination of how the energy difference associated with primary charge separation correlates with motions of protein and solvent molecules around the exchanging chromophores. Our analyses show that the interconversion between the fast (hundreds of femtoseconds) and slower (a few picoseconds) regimes is driven by dynamic rearrangements of water molecules within the protein matrix, including the water channels that regulate access to the oxygen-evolving complex. These findings clarify the molecular basis of the hierarchical timescales in primary charge transfer, highlighting the distinct and complementary roles of both protein and solvent dynamics in shaping electron transfer kinetics.*"

6. The Berendsen barostat is known to not reproduce the proper ensemble. Why was the Rahman-Parrinello barostat not use and what are the potential consequences? What was the simulation temperature and pressure?

Although the Berendsen barostat does not rigorously sample the NPT ensemble, it is widely recognized for its numerical stability and robustness. We employed it here to ensure consistency with previous MD simulations of the PSII system, which were all performed with Amber [J. Am. Chem. Soc. 142 (42), 18174-18190, 2020; Angew. Chem. Int. Ed. 62, e202200356, 2022, Angew. Chem. Int. Ed. 62, e202216276, 2023]. These studies have already documented extensively the stability of the system along the trajectories, including the membrane and the total hydration [see J. Am. Chem. Soc. 144, 22035-22050, 2022]. Key averaged properties such as mean pressure and density remain stable. Given the very large size and complexity of PSII, it was essential to prioritize numerical stability to ensure the reliability of the simulations. We acknowledge that the Berendsen barostat modifies volume and pressure fluctuations. However, these effects primarily impact slow collective modes of the membrane–protein complex, which are largely unrelated to the ultrafast charge and energy transfer processes discussed here, occurring on the sub-picosecond timescale

The simulation pressure and temperature were 1 bar and 303 K, respectively. We apologize for the oversight: the simulation temperature was missing in the submitted version, and we have now included the correct value.

7. The predicted spectra in Figure 7B match experiment poorly. How does the lack of predictive power of the used model affect the other properties and the conclusions?

We hypothesize that the reviewer is referring to the fact that the peak at 680 nm in our simulation is narrower than in the experiment. This, however, is not surprising, as the calculated spectrum obtained with the perturbed matrix method was applied here only to three out of at least eight chromophores typically absorbing at 680 nm in isolated RC complexes (four core Chl a, two peripheral Chl a, and two pheophytins). The underestimation of the width can be rationalized by the fact that our model neglects contributions from the remaining pigments, which are known to broaden the spectral distribution through excitonic interactions, and that quantum vibrational effects are not included.

However, our aim here was not to perform a detailed comparison with experiments, as, for example, we previously and successfully did for CP43 [Bhattacharjee et al. Chem. Sci. 2024,

15.19, 7269-7284], but rather to establish the relative intensities of ChlD1 and SP at 680 nm for defining the starting populations in the kinetic model. In the case of CP43, where we considered 14 Chl a pigments, there was a close match with the experimental line shape. Accurate modeling of the absorption spectrum here would require more extensive calculations and is beyond the scope of the present study. We recognize that, as pointed out by the reviewer, by presenting in Figure 7 both the calculated and the experimental spectra it seems that we aim for a direct and quantitative comparison. Therefore, we removed the experimental spectrum from Fig. 7. At the same time, we now acknowledge that our computed spectrum is narrower than the experimental one explaining the origin of this discrepancy. Regarding the predictive power of our computational approach, as stated at the beginning of the "Kinetic Model" section, we used the reproduction of the apparent lifetimes as an indicator of the reliability of our calculations.

The new paragraph in the SI reads as follows (page 5):

"*It should be noted that the absorption spectrum calculated in the present case includes only three out of at least eight chromophores typically absorbing at 680 nm in isolated RC complexes (four core chlorophylls a, two peripheral chlorophylls a, and two pheophytins). The fact that our model neglects contributions from the remaining pigments, which are known to broaden the spectral distribution through excitonic interactions, and that quantum vibrational effects are not included, is expected to lead to an underestimation of the width. However, our aim here is not to perform a detailed comparison with experiment, as was successfully done, for example, for the CP43 complex [Bhattacharjee et al. Chem. Sci. 2024, 15.19, 7269-7284] which contains 14 chlorophylls, but rather to establish the relative intensities of ChlD1 and SP at 680 nm for defining the starting populations in the kinetic model.*"

8. Provide references for exchange correlation functional and basis set in section 1.1.

As suggested by the reviewer, we have added proper references to the functional and basis set used.

9. Provide the calculated TrESP charges in the SI.

As suggested by the reviewer, we have added two tables with TrESP data for ChlD1 (Table S4, SI) and SP (Table S5, SI) in the supporting information.

10. Report the used transition dipoles in de PDA.

As suggested by the reviewer, we have added the transition dipole magnitude in section 1.1 of the SI as follows (page 3):

"The calculated magnitudes for transition dipoles are 5.83 D and 7.36 D for ChlD1 and SP, respectively. "

11. Repeat the values of the couplings given in the main in the table of SI1.1 for simplicity for the reader.

As suggested by the reviewer, we have added the coupling magnitudes to Table S1 in section 1.1 of the supporting information..

12. Why were different DFT functionals used for the TrESP and energy calculations?

We thank the referee for pointing out this discrepancy in the Methods section. The inconsistency arises from a mere careless mistake: the actual functional and basis set used are ωB97X and Def2-TZVP, respectively, for both the TrESP and PMM energy calculations. The latter are indeed the same calculations reported in our previous publication [Capone et al. Angew. Chem. Int. Ed. 62, e202216276, 2023]. We apologize for the error in the original version. The supplementary section has been updated providing the appropriate references for the functional and basis set.

13. Provide an explicit equation for the transition energy.

As suggested by the reviewer, we modified and expanded section 1.2 in the Supporting Information also explicitly adding the expression for the transition energy. The expression for the transition energy was also added to the main text (page 4).

Reviewer #2:

The authors investigate charge separation (CS) in the Photosystem II (PSII) reaction center, focusing on the ChlD1^{+} pathway through the ChlD1^{+}PheoD1^{-} intermediate. They propose that structural changes in the protein and the surrounding water particularly dynamic water channels near the oxygen-evolving complex (OEC) help explain the different timescales observed in the two steps CS pathway: RC --> ChlD1^{+}PheoD1^{-} --> SP^{+}PheoD1^{-}

To address ongoing debates about CS timescales, they use a multiscale computational approach combining molecular dynamics, QM/MM and the Perturbed Matrix Method (PMM). This allows them to model how environmental flexibility influences CS and they also apply Förster theory to estimate excitation energy transfer within the reaction center. Their model provides insight into how CS is modulated by water channel and protein conformational changes, supporting a refined interpretation of parallel pathways.

While the work is scientifically sound and likely publishable, I have some major concerns that the authors should address before acceptance:

1. Why did the authors choose to use TrESP exciton coupling to calculate couplings between the pigment pairs within the reaction center, which involves strong charge separation character? I disagree with the statement that "short-range coupling originating from the orbital overlap of frontier molecular orbitals might not have major contributions to the overall excitonic coupling. Hence, the short-range effects are neglected." When modeling coupling between charge separation states, a diabatization scheme should be considered. As validation, the authors could perform TD-DFT calculations in gas-phase on the special pair to compute the full coupling V = 1/2 (E1 - E2), where E1 and E2 are the first two excited state energies and then compare this to TrESP coupling values. I expect significant short-range orbital overlap effects in the coupling that would impact the kinetics.

We agree with the reviewer that within the special pair, where the two pigments are strongly pi–pi stacked, short-range orbital overlap makes a major contribution to the excitonic coupling, and approaches such as TD-DFT exciton splitting would capture these effects well. However, we focus here on the coupling between different pigments, namely between Chl_D1 and the special pair (the latter considered as a single complex), between which there is no charge transfer, to calculate energy transfer. In this case, the pigments adopt an edge-to-face geometry, with the methyl groups of Chl_D1 oriented toward the special pair (Fig. 2A), and the centroid-to-centroid distance is significantly larger than that within the special pair. These structural features strongly reduce orbital overlap. Furthermore, the electronic coupling for electron transfer is proportional to the orbital overlap, whereas that for energy transfer is proportional to the square of the overlap. For all these reasons, short-range contribution to Chl_D1–SP coupling is expected to be minimal and the TrESP approach is expected to provide an appropriate estimate of the energy transfer coupling in our system. We have better substantiated our choice to neglect short range effects (page 3):

"*Based on the edge-to-face orientation of ChlD1 and SP and on the centroid-to-centroid distance (1.04 nm), the short-range coupling originating from the orbital overlap of frontier molecular orbitals might not have major contributions to the overall excitonic coupling. Hence, the short-range effects are neglected.*"

2. Why was the Förster rate equation used to model population kinetics involving electron transfer? Marcus theory should be applied instead, as it better accounts for strong charge separation coupling within a diabatization framework.

We would like to clarify that Förster theory is not used for any calculations involving electron transfer reactions. It is applied solely to compute the energy transfer rate between ChlD1* and SP forming ChlD1 and SP* (and vice versa), as described in the main text and Figure 1B. We have carefully reviewed the manuscript and did not find any inappropriate references to Förster theory in the context of electron transfer.

3. Although the authors have demonstrated the effect of the protein on the charge separation state and its kinetics using a single structure (J. Am. Chem. Soc. 142, 18174–18190 (2020)) and dynamics with the PMM method (Angew. Chem. Int. Ed. 62, e202216276 (2023)), I wonder what new insights this study provides beyond the comprehensive analysis of the different charge separation pathways within the PSII-RC.

The main insight of the present study is the mechanistic interpretation of the bi-exponential behavior observed in the primary charge separation kinetics. As the reviewer notes, in our previous work (Angew. Chem. Int. Ed. 62, e202216276, 2023), we reported a bi-exponential kinetics for this reaction, but without being able to identify the precise atomistic origin of these observations. In the current study, we have uncovered and are reporting the molecular origin for this behavior, attributing it to water-modulated conformational heterogeneity within the PSII reaction center. To the best of our knowledge, this is the first time that the distinct roles of both the protein and the solvent in modulating electron transfer kinetics have been explicitly identified and characterized in this context. In addition, our findings provide a reconciling scenario for the bi-exponential kinetics observed experimentally for the first charge-transfer step with atomistic-level mechanisms.

We have now rephrases part of the conclusions to highlight this point (page 10):

"We propose that the ChlD1+ pathway, i.e. the direct reduction of PheoD1 by the excited ChlD1, is the dominant charge-separation route, and that the bi-exponential kinetics instead reflect conformational heterogeneity within this pathway. This conclusion is supported by a detailed examination of how the energy difference associated with primary charge separation correlates with motions of protein and solvent molecules around the exchanging chromophores. Our analyses show that the interconversion between the fast (hundreds of femtoseconds) and slower (a few picoseconds) regimes is driven by dynamic rearrangements of water molecules within the protein matrix, including the water channels that regulate access to the oxygen-evolving complex. These findings clarify the molecular basis of the hierarchical timescales in primary charge transfer, highlighting the distinct and complementary roles of both protein and solvent dynamics in shaping electron transfer kinetics."

4. I am curious about the mechanism that enables such a long-distance charge separation state between SP^+ and $PheoD1^-$. Is this driven by strong HOMO-LUMO overlap or are other factors involved?

New: In the present paper, we do not focus on the long-range CT between SP and PheoD1 but rather on the short-range one between ChlD1 and PheoD1. To try to respond to the referee's comment, we can expand on the results we presented in our previous paper in which the long-range CT was discussed. For such a long-range charge transfer, the HOMO-LUMO

overlap is negligible and is thus not expected to be the main driving force. Our previous calculations (Capone et al. Angew. Chem. Int. Ed. 62, e202216276, 2023) showed that the mean transition energy (TE) associated with the long-range CT is close to zero, i.e. reactants and products are nearly degenerate. At the same time, the reaction free energy (ΔG) is highly negative, indicating a strong thermodynamic driving force. Both TE and ΔG are strongly tuned by the protein environment, which modulates the pigment redox levels and electrostatics to stabilize the charge-separated state and suppress recombination. Thus, the existence of a long-range CT is not explained by strong orbital overlap, but rather by the environmental tuning that makes the charge separation energetically favorable.

Minor points:

I think the crystal structure (3WU2) does not show the full PSII because it is missing the main antenna complex, LHCII. It looks like it only includes the PSII core (CP43 and CP47) and the reaction center (D1 and D2) complexes. I suggest the authors use PSII-RC instead of PSII when referring to the reaction center.

The computational model does not include only the four proteins mentioned above, but all 20 proteins and all cofactors of the monomer complex. The use of the term “PSII” to refer to the complete system without the associated LHCII is standard practice in the field, including in the crystallography papers themselves.

Reviewer #3:

In this manuscript the authors present a well-executed and conceptually strong study that addresses a long-standing question in photosynthetic charge-separation kinetics. The authors combine ultrafast kinetic modeling with detailed molecular dynamics simulations using the MD–Perturbed Matrix Method to elucidate the role of protein–solvent dynamics, particularly water-channel reorganization, in governing the observed multi-exponential behavior of primary charge separation.

The work is of high quality in several respects:

1) Scientific significance: The study provides a unifying explanation for disparate experimental observations and resolves a persistent debate regarding the origin of multiple time constants in Photosystem II.

2) Methodological rigor: The combination of extended atomistic MD simulations, hybrid quantum/classical treatment, and numerical kinetic modeling is thorough and well justified. The MD-PMM framework is applied appropriately, and the details provided inspire confidence in the reproducibility and validity of the results.

3) Clarity of presentation: The manuscript is well written, with the Introduction and Discussion clearly framing the context and implications of the findings. Figures and tables are clear, relevant, and informative.

4) Quality of results: The agreement between the simulated apparent lifetimes and experimental measurements is impressive. The link between structural fluctuations of water channels and kinetic heterogeneity is convincingly demonstrated.

The conclusions are fully supported by the data, and the manuscript advances our mechanistic understanding of ultrafast charge separation in a key photosynthetic complex. It will be of interest to researchers in photosynthesis, biophysics, and theoretical/computational chemistry. I strongly support publication of this manuscript in its present form. The study is a valuable contribution to the field and meets the high standards of the journal.

We thank the referee for her/his very positive feedback on our work.

Reviewer #4:

This paper provides an important insight. The authors present a comprehensive model of primary charge separation, based on their theoretical calculations in reference to recent experimental results.
The work is worthy of publication. However, the problem itself is complex, and the available experimental results are often limited and partial. The reviewer would like to request further discussion as outlined below:

To what extent, and with what accuracy, can the current theoretical methods employed here be considered reliable? What are their limits of applicability? Convincing references to past literature, if available, are needed. This is critical in order to avoid arbitrary adjustments of the calculated results, given that experimental results vary from case to case.

We thank the reviewer for giving us the opportunity to highlight the strength of the PMM approach in reproducing and explaining the underlying mechanisms of many experimental observables in complex systems, such as redox potentials, side-chain pK_a values, IR spectra, UV–vis absorption spectra, and electron transfer rates. Relevant references include: Bortolotti et al. J. Am. Chem. Soc. 2012, 134(33), 13670-13678; Zanetti-Polzi et al. J. Phys. Chem. Lett. 2017, 8, 3321; Davis et al. Chem. Sci. 9.48 2018, 9002; .Zanetti-Polzi et al. J. Phys. Chem. Lett. 2021, 12, 4195; Li et al. J. Am. Chem. Soc. 2023, 145, 13232; Capone et al. Angew. Chem., Int. Ed. 2023, 62, E202216276; Capone et al. ACS Catal. 2024, 14, 16488–16496; Bhattacharjee et al. Chem. Sci. 2024, 15.19, 7269-7284.

The MD-PMM approach offers a significant advantage over Non-Adiabatic Mixed Quantum–Classical (NA-MQC) dynamics, as it enables extensive sampling of the conformational space and thus captures the coupling between ET and protein/solvent dynamics, especially over long timescales. Unlike NA-MQC dynamics, however, MD-PMM does not explicitly propagate the electronic wave function, which becomes critical in situations where the dominant charge-transfer mechanism involves ultrafast intramolecular relaxations. In PSII, however, conformational flexibility of the environment plays a key role in modulating RC properties, making PMM particularly well suited.

We have now emphasized more clearly in the Introduction and Supporting Information the capability of our method to reproduce experimental observables in complex systems, as well as its main limitations.

In the Introduction (page 3):

"*This approach has already been reliably used to reproduce and explain the underlying molecular mechanisms of many experimental observables in complex systems, including redox potentials, side-chain pKa values, infrared and UV–vis absorption spectra, and electron transfer rates [Bortolotti et al. J. Am. Chem. Soc. 2012, 134(33), 13670-13678; Zanetti-Polzi et al. J. Phys. Chem. Lett. 2017, 8, 3321; Zanetti-Polzi et al. J. Phys. Chem. Lett. 2021, 12, 4195; Li et al. J. Am. Chem. Soc. 2023, 145, 13232; Capone et al. Angew. Chem., Int. Ed. 2023, 62, E202216276; Capone et al. ACS Catal. 2024, 14, 16488–16496; Bhattacharjee et al. Chem. Sci. 2024, 15.19, 7269-7284]*"

In the SI (page 3):

"*The MD-PMM approach offers an advantage over Non-Adiabatic Mixed Quantum–Classical (NA-MQC) dynamics methods, as it enables extensive sampling of the conformational space*

and thus captures the coupling between electron transfer (ET) and protein/solvent dynamics, especially over long timescales. Unlike NA-MQC dynamics, however, MD-PMM does not explicitly propagate the electronic wave function, which becomes critical in situations where the dominant charge-transfer mechanism involves ultrafast intramolecular relaxations. In Photosystem II, however, conformational flexibility of the environment plays a key role in modulating RC properties, making the PMM particularly well suited."

Related to the above, the OEC is well known for its challenging spin states. In the present problem, which also involves radical states, how accurate are the theoretical methods?

The situation in the OEC is distinct because the problem there is the description of the magnetic exchange coupling among "strongly interacting" (in the context of an effective spin Hamiltonian) high-spin Mn ions, which lead to closely spaced spin multiplets. In the present problem the radical states (if directly computed in a diradical form) would be well-localized and non-interacting, therefore the question of spin-coupling and spin-state energetics overall does not arise.

Although the following question is somewhat philosophical, the reviewer would still like to know the authors' view. Figure 1B shows three possible pathways, and the authors focused on the ChlD1$^+$ pathway. Fundamentally, the phenomenon involves the selection of quantum mechanical states. Therefore, choosing a pathway corresponds to choosing the weight or amplitude of the states. Is this weight beyond the scope of the current method?

This is a very interesting point of view and we are excited that the Reviewer anticipates some future work that is currently under development. In short, we believe that a description in terms of selection of quantum mechanical states is indeed achievable if all possibilities are simultaneously computed, but the way to do this is not with a DFT-based approach but by constructing state-averaged multireference wave functions that are still embedded in the electrostatic field of the protein. We have already presented the first step toward this goal in the form of a multireference *ab initio* treatment of single pigments in the RC (see Drosou, M., Bhattacharjee, S. & Pantazis, D. A. "Combined Multireference–Multiscale Approach to the Description of Photosynthetic Reaction Centers". *J. Chem. Theory Comput.* **20**, 7210-7226, (2024)) and we are currently extending this to pairs of pigments. Once the multireference description of at least four pigments becomes possible, then the above type of analysis will be directly accessible.

Minor questions

In the last phrase of Protein effect: "..., rather than a unique quantum center," the meaning is unclear. Could the authors clarify?

To clarify the meaning of the sentence mentioned by the reviewer, we rephrased it as follows (page 8):

"Importantly, we were able to determine these contributions in this work by treating the two CT partners as separate quantum centers (i.e., by performing separate QM calculations on SP and ChlD1, see section 1.2 in the Supporting Information) rather than as a complex."

In addition, we modified and expanded section 1.2 in the Supporting Information to further clarify the procedure used.

In Figure 4, panel A, the right-side graph is aligned with the arrays beside the slow-ET, although it should correspond to the fast-ET. Could the authors check this?

We thank the reviewer for underlining the ambiguity of interpretation in the figure. We changed the appearance for Fig.4 using the same portion of MD in both the zoom panels to prevent misunderstanding due to the alignment. We hope that the changes made help to visually clarify the different effects of the protein and the solvent on the TE.

Reviewer #1 (Remarks to the Author):

The authors have revised the manuscript based on the feedback from all reviewers. While most points improved a few misconceptions sneaked in. In the answer to the question regarding coherent components to the EET the authors now (in their answer and the manuscript) claim that a coherent component would speed up the transfer. This is a misconception. Equation (1) in the manuscript is known to predict too fast transfer as the coherent limit is approached (see for example 10.1063/5.0136652, but undoubtedly this is also discussed in much older literature by for example Silbey and Renger.) As the coupling increase the cross-over leads to a slower J dependence on the coupling instead of the J^2 one. Förster transfer provides an upper limit NOT a lower limit. A coherent component would slow it down as the next fourth-order perturbation theory has the opposite sign of the second-order. It would be better to demonstrate that the present parameters are in the regime where Eq. (1) is applicable for example by comparing with other methods (as also proposed by another reviewer).

We thank the reviewer for carefully pointing out the role of coherence in energy-transfer kinetics.

In response, we have substantially revised the methodology for obtaining energy transfer rates. Instead of the Förster rate equation, valid in the incoherent (very weak electronic coupling) limit, we now have employed numerically exact quantum dynamics treatment using time-dependent density matrix renormalization group (TD-DMRG) method at 300K (see section 1 of the revised Supplementary Information, SI). With this approach, both electronic coherence and system-bath interactions are treated on equal footing. As a result, any coherent effects in energy transfer dynamics are accounted for. In addition, we have replaced the earlier dimer model (SP-ChlD1) with a more widely utilized trimer model (PD1-PD2-ChlD1), with the system-bath interaction modeled *via* the spectral density accounting for both pigment-protein interaction, as well as intramolecular effects, which was measured experimentally by R. Van Grondelle, J. Dekker and coworkers [R. van Grondelle et al., *Proc. Natl. Acad. Sci. U. S. A.*, **1998**, 95, 6128-6133]. In the TD-DMRG quantum simulation, we used TrESP-based couplings taken from the literature, which were scaled to account for short-range effects within the SP and for environmental screening effects [Renger and coworkers, *Biophys. J.* 95.1, **2008**, 105-119]. More details about the coupling parameters are provided in our response to Reviewer 2, and suitable details have been incorporated into the revised version of the SI (section 1.1).

The TD-DMRG simulation shows that the coherent character of the population transfer is moderate, although not completely suppressed. The results, shown in Figure 2 of the revised version of the manuscript, reveal a gradual decay of coherence between ChlD1

and the SP subunits. Among these, the ChlD1-PD2 coherence is higher, owing to the slightly larger coupling. Furthermore, the strong excitonic coupling V between PD1 and PD2 leads to the gradual buildup of a modest but persistent coherence between them.

The population decay of ChlD1* obtained from the quantum dynamics simulation was fitted using a reversible two-state kinetic model, enabling extraction of the forward (SP* → ChlD1*) and backward (ChlD1* → SP*) time constants: $\tau_{forward}$=296 fs and $\tau_{backward}$=928 fs, corresponding to a phenomenological relaxation time of 224 fs.

Overall, the main consequence of including coherence effects in the EET description and the use of the new set of electronic couplings is that the EET observed time constant is now on the 200 fs timescale, rather than 90 fs, as was obtained in the previous calculations. Nonetheless, the overall conclusions of the study remain unchanged.

We have now thoroughly revised the section describing the EET calculations and the one reporting results on the global kinetic model.

The authors now report the simulation temperature and pressure. The used constants for the thermostat+barostat (time-scale and compressibility) are not reported. I strongly recommend that the authors ensure that the results will be reproducible.

We thank the Reviewer for carefully checking the parameters required for proper reproducibility of our work. The Computational Methods section already stated the timescale parameters for the thermostat (1 ps) and barostat (2 ps). We have now also included the compressibility parameter used in the simulations (4.5 × 10^{-5} bar^{-1}). In addition, all files required to restart the simulations (coordinates, topology, and AMBER input files) have been uploaded as additional supplementary information.

Main text, page 12:

"The temperature of the MD simulations was set to 303 K and controlled using Langevin dynamics, with a collision frequency of 5 ps^{-1} during the heating steps and 1 ps^{-1} during the constant-temperature phases. Pressure was regulated anisotropically using the Berendsen barostat, with a relaxation time of 2 ps and a target pressure of 1 bar. The isothermal compressibility was set to 4.5 × 10^{-5} bar^{-1}."

The authors removed the experimental data provided for comparizon. I would recommend not hiding it, but state clearly why the agreement can be considered sufficient for the purpose of the paper. I would avoid removing data which could be seen as cherry picking.

We agree with the Reviewer's assessment and have retained the experimental reference in the figure. We have also added a discussion on why the agreement can be considered sufficient for the purpose of the paper (page 10). Additionally, we have updated the initial populations used in the global kinetic scheme in agreement with the new calculations performed in response to the second Reviewer's request regarding the nature of the SP-ChlD1 trimer's excited electronic states. These new populations were obtained based on the participation ratios of the trimer chromophores (SP and ChlD1) across the first three excited states, yielding values of 49% for SP and 51% for ChlD1. These updates are now reflected in the main text (page 10), with the underlying electronic state calculations provided in the Supporting Information (section 3.1).

Main text, page 10:

"To reproduce the experimental pump, the initial populations are determined from the delocalization of the first two excited states, as obtained from participation ratio (PR) analysis, and from the corresponding absorption intensity on the red side of the main peak, calculated within the MD-PMM approach (see Fig. 7B) providing 49% ChlD1 (red) and 51% SP* (orange); for the details of the calculations of the absorption spectrum and of the PR to the first three excited states see section 3 of the SI. These were then weighted with the relative populations of the two conformational states (namely 40% for the "slow-ET" regime and 60\% for "fast-ET" regime, see Fig. 3). The resulting starting populations are: P(ChlD1*, fast-ET)=29%, P(ChlD1*, slow-ET)=20%, P(SP*, fast-ET)=31% and P(SP*, slow-ET)=20%. It should be noted that the absorption spectrum calculated in the present case includes only three out of at least eight chromophores typically absorbing at 680 nm in isolated RC complexes (four core chlorophylls a, two peripheral chlorophylls a, and two pheophytins). The fact that our model neglects contributions from the remaining pigments, which are known to broaden the spectral distribution through excitonic interactions, and that quantum vibrational effects are not included, is expected to lead to an underestimation of the width. However, our aim here is not to perform a detailed comparison with experiment, as was successfully done, for example, for the CP43 complex which contains 14 chlorophylls, but rather to establish the relative intensities of ChlD1 and SP at 680 nm for defining the starting populations in the kinetic model."*

Reviewer #2 (Remarks to the Author):

The study is interesting and technically well executed, and I appreciate that the revised version attempts to address the reviewers' earlier comments. However, some fundamental methodological limitations remain, which in my view reduce the overall novelty and suitability of the work for Nature Communications.

The coupling between ChlD1 and SP* is still evaluated using the TrESP approach, assuming negligible charge-transfer or overlap contributions. In the PSII reaction center, however, exciton and charge-transfer mixing are commonly known to occur, and this assumption may not be fully justified for confirming the bi-exponential excitation pathways. To verify that short-range orbital overlap and charge-transfer effects are indeed minor, I would still recommend performing TD-DFT calculations (e.g., exciton splitting or CT character analysis) on representative pigment dimers. This would provide a firmer validation of the coupling strengths used in the kinetic model.

As suggested by the Reviewer, we performed gas-phase quantum chemical calculations on the pigments involved in the reaction to assess the relevance of both charge-transfer (CT) mixing and short-range effects.

CT character of the excited states. Specifically, concerning the first point, we analyzed the CT character of the first 5 excited states of the trimer using TheoDORE, which enables quantitative decomposition of excited states into local and charge-transfer contributions. The results, reported below and included in the revised version of the Supporting Information (section 3.1), show that no charge-transfer character is present between the special pair and ChlD1 in the first 5 excited states. To further characterize the nature of the excitations, we also computed the participation ratio (PR), which quantifies the exciton delocalization between the SP and ChlD1 subunits within the trimer (see next Table). These last data were used to update the initial populations used in the global kinetic scheme.

**Excited state**	**ΔE (eV)**	**CT character**	**PR**
S1	1.873	0.002	1.663
S2	1.884	0.001	1.044
S3	1.911	0.002	1.611
S4	2.385	0.003	1.049
S5	2.393	0.001	1.021

Short-range contributions to the couplings. Concerning the second point, we followed the suggestion provided by the Reviewer in the first round of review and used a diabatization scheme to compute the full couplings. In the revised framework, the special pair is no longer described as a single unit coupled to ChlD1; instead, the three chlorophylls are treated separately. This choice is motivated by the requirements of quantum dynamical simulations of

the EET process (TD-TDMRG) performed to account for possible coherence effects (see answers to Reviewer 1).

For the PD1-PD2 pair, short-range effects were thoroughly investigated by Renger and coworkers using multiscale simulations and a diabatization scheme combining the Fragment Charge Difference (FCD) and the Fragment Excitation Difference (FED) methods [T. Renger and coworkers, *J. Phys. Chem. Lett.* **2023**, 14, 11758−11767]. For the ChlD1-PD1 and ChlD1-PD2 pairs, we computed the full couplings (J1 and J2, respectively) using the FED scheme as implemented in Q-Chem. Such a scheme is appropriate when no relevant CT character between the involved states is present, as is the case here.

We performed these calculations on 11 structures extracted at regular intervals along the MD simulation and, for each structure, we compared the full coupling between ChlD1 and PD1 and between ChlD1 and PD2 with that obtained using the TrESP approach. The results, reported below and included in the revised version of the Supporting Information (section 1.1), show that the TrESP approach underestimates the couplings by 3-8%, suggesting a minor contribution from short-range effects. The calculations of the couplings for the individual pigments also allowed us to verify that our results are in good agreement with previous literature data. In fact, our couplings are slightly larger than previously reported values obtained with the TrESP approach. However, the latter [Renger and coworkers, *Biophys. J.* 95.1, **2008**, 105-119] include a scaling factor accounting for environmental effects.

Based on the above results on the minor contribution of short-range effects for the ChlD1-PD1 and ChlD1-PD2 pairs, we used in the new TD-DMRG quantum simulations of the EET process the set of couplings provided by Renger and coworkers for consistency with previous works: V = 150 cm^{-1} for the PD1-PD2 pair, obtained from fits of optical spectra and empirically incorporating the environmental and short-range effects; J_1 = -42 cm^{-1} and J_2 = -56 cm^{-1} for the PD1-ChlD1 and PD2-ChlD1 pairs, respectively, obtained using the TrESP approach with a rescaling procedure to account for environmental effects.

**Timestep(ns)**	**$\lvert J_1^{TrESP}\rvert (cm^{-1})$**	**$\lvert J_1^{FED}\rvert (cm^{-1})$**	**$\lvert J_2^{TrESP}\rvert (cm^{-1})$**	**$\lvert J_2^{FED}\rvert (cm^{-1})$**
0	61.4	66.2	79.0	81.4
20	58.8	64.5	64.5	65.3
40	59.0	62.9	68.9	70.3
60	57.9	62.5	74.6	77.4
80	57.7	62.0	86.3	90.4
100	64.0	69.3	77.0	80.2
120	61.4	67.3	73.6	76.2
140	67.3	71.6	69.3	71.3
160	64.4	69.5	80.4	83.9
180	59.8	64.1	69.2	70.2
200	68.1	73.8	68.1	68.2
**Average**	**61.8 (1.1)**	**66.7 (1.2)**	**74.0 (2.0)**	**75.9 (2.3)**

In addition, the PMM-MD methodology remains based on classical molecular dynamics sampling, which cannot capture nonadiabatic transitions or electronic coherence that likely influence ultrafast charge-separation dynamics. While PMM is valuable for\ describing environmental fluctuations, it lacks the explicit quantum treatment of electronic motion available in mixed quantum-classical (MQC) approaches. This limitation should be discussed more explicitly, as it constrains both the mechanistic interpretation and the generality of the conclusions.

Following the referee's suggestion, we now explicitly state in the introduction that the MD-PMM approach lacks the explicitly propagation of the electronic wavefunction. Concerning the possible role of electronic coherence effects, which are not included in PMM-MD simulations, recent experimental and theoretical work has demonstrated that coherent electronic dynamics associated with the ChlD1–PheoD1 charge-transfer process is completely suppressed at room temperature [Jha et al. *Science advances* 10.10 **2024**: eadk1312]. This result indicates that coherence effects do not play a significant role in this specific charge-transfer step and can therefore be safely neglected within the present modeling framework. We have now inserted the following discussion in the Introduction (page 3):

"The extensive sampling of the conformational space achievable with the MD-PMM approach enables the capture of the coupling between intermolecular electron transfer and protein/solvent dynamics over long timescales. However, unlike non-adiabatic mixed quantum–classical (NA-MQC) dynamics methods, MD-PMM does not explicitly propagate the electronic wavefunction, which may become critical in systems dominated by ultrafast intramolecular relaxation processes. Moreover, it does not explicitly treat electronic coherence. In the case of the ET from ChlD1 to PheoD1 in the reaction center of PSII, however, the process is intermolecular and is expected to be strongly modulated by protein and solvent fluctuations. Under these conditions, and given the experimentally demonstrated suppression of electronic coherence at physiological temperatures [Jha et al. Science advances 10.10 (2024): eadk1312], the MD-PMM approach provides an appropriate and physically justified description of the charge-transfer process."

Overall, the manuscript presents a careful and informative study, but the methodological framework remains semi-classical, and the results largely represent an incremental extension of previous work by the same group. Thus, although the topic is of clear scientific interest, in my view, the manuscript may not yet reach the level of conceptual advance expected for Nature Communications.

We thank the Reviewer for this assessment. The concern about the semi-classical nature of MD-PMM has now been addressed in our response above and in the revised manuscript: we have explicitly acknowledged this limitation, discussed its scope, and provided justification for why it does not affect the conclusions for the specific charge-transfer step modeled here (CT1),

based on the experimentally demonstrated suppression of electronic coherence at room temperature. Regarding the EET step, we have now replaced the Förster treatment with numerically exact TD-DMRG quantum dynamics, which fully accounts for coherence effects.

On the broader question of novelty, we would like to respectfully highlight the key conceptual contribution of this work. Previous studies have interpreted the multi-exponential charge separation kinetics in PSII in terms of parallel pathways involving chemically distinct radical pair intermediates. Our work proposes a fundamentally different interpretation: the multi-exponential behavior arises from a single charge-separation pathway ($ChlD1^{+}PheoD1^{-}$) modulated by water-driven conformational heterogeneity. This mechanistic reinterpretation, directly connecting ultrafast charge separation to the structural dynamics of the water channels that regulate the oxygen-evolving complex, represents a conceptual advance beyond incremental extension. We believe this distinction, and its implications for understanding the coupling between charge separation and water oxidation in PSII, is of broad interest to the readership of Nature Communications.
